# Evolution of plasticity in production and transgenerational inheritance of small RNAs under dynamic environmental conditions

**DOI:** 10.1371/journal.pgen.1009581

**Published:** 2021-05-26

**Authors:** Willian T. A. F. Silva, Sarah P. Otto, Simone Immler

**Affiliations:** 1 Department of Evolutionary Biology, Uppsala University, Uppsala, Sweden; 2 Department of Zoology, University of British Columbia, Vancouver, Canada; 3 School of Biological Sciences, University of East Anglia, Norwich, United Kingdom; University of Rochester, UNITED STATES

## Abstract

In a changing environment, small RNAs (sRNAs) play an important role in the post-transcriptional regulation of gene expression and can vary in abundance depending on the conditions experienced by an individual (phenotypic plasticity) and its parents (non-genetic inheritance). Many sRNAs are unusual in that they can be produced in two ways, either using genomic DNA as the template (primary sRNAs) or existing sRNAs as the template (secondary sRNAs). Thus, organisms can evolve rapid plastic responses to their current environment by adjusting the amplification rate of sRNA templates. sRNA levels can also be transmitted transgenerationally by the direct transfer of either sRNAs or the proteins involved in amplification. Theory is needed to describe the selective forces acting on sRNA levels, accounting for the dual nature of sRNAs as regulatory elements and templates for amplification and for the potential to transmit sRNAs and their amplification agents to offspring. Here, we develop a model to study the dynamics of sRNA production and inheritance in a fluctuating environment. We tested the selective advantage of mutants capable of sRNA-mediated phenotypic plasticity within resident populations with fixed levels of sRNA transcription. Even when the resident was allowed to evolve an optimal constant rate of sRNA production, plastic amplification rates capable of responding to environmental conditions were favored. Mechanisms allowing sRNA transcripts or amplification agents to be inherited were favored primarily when parents and offspring face similar environments and when selection acts before the optimal level of sRNA can be reached within the organism. Our study provides a clear set of testable predictions for the evolution of sRNA-related mechanisms of phenotypic plasticity and transgenerational inheritance.

## Author summary

Small RNAs (sRNA) are produced by a wide range of organisms, from bacteria to plants and animals. These molecules are involved in the response to environmental stress (e.g., temperature, pathogens) and can be transmitted across generations. We developed a model to explore the dynamics of sRNA production (phenotypic plasticity) and inheritance in a fluctuating environment. We tested whether different sRNA mutants can invade a population where individuals produce sRNA at a constant optimal transcription rate. In our simulations, plastic amplification rates capable of responding to environmental conditions were favored and the transmission of sRNA transcripts or amplification agents across generations was particularly advantageous when parents and offspring faced similar environments. sRNA amplification alone is not favored except when optimal sRNA levels are not reached within a generation. Our model provides novel predictions for the molecular mechanisms of sRNA production and guidance for future empirical studies on mutations that impair the mechanisms of sRNA production and their fitness consequences.

## Introduction

Faced with a continually changing environment, organisms have evolved to respond to their environment (phenotypic plasticity) and to transfer information about environmental conditions from parents to offspring (non-genetic inheritance), influencing a broad array of ecological and evolutionary processes [1–6]. Several studies in animals and plants suggest that some non-genetic factors inherited across generations have the potential to be beneficial to both parents and their offspring [7,8]. A key step in assessing the role of non-genetic inheritance in adaptive processes is to improve our understanding of the different mechanisms involved. Three important factors currently thought to play a role in phenotypic plasticity and non-genetic inheritance include DNA methylation, chromatin structure modifications, and certain families of RNAs [6,9] (we follow [3] in using "non-genetic" inheritance to encompass a broad array of mechanisms by which parents can influence offspring, including epigenetic inheritance). In particular, small RNAs (sRNAs) have been shown to play a potentially important role in transferring information about parental conditions to offspring due to their diversity in function [10] and their apparently rapid evolution [11,12]. Here, we develop a model to assess how changing environmental conditions drive the evolution of plasticity and inheritance of sRNA levels.

sRNAs are short (<200 nucleotides) non-coding RNAs that have been recently discovered and described across the tree of life, from bacteria [11,13,14] to plants [15–17] and animals [18–20]. The functions vary for different sRNA families and across taxa. In animals, two key functions are relevant for their potential role in adaptive processes, namely the post-transcriptional regulation of gene expression [21,22] and the preservation of genome integrity through the repression of parasitic DNA elements such as transposable elements (TEs) [23]. Two animal sRNA families that are particularly associated with these two functions are microRNAs (miRNA) and Piwi-interacting RNAs (piRNA) [24]. miRNAs have been found to regulate adipocyte differentiation in humans [25], hematopoietic differentiation in house mice *Mus musculus* [26], cell proliferation and apoptosis in *Drosophila* [27,28], the maternal-to-zygotic transition in zebrafish *Danio rerio* [29], and several other developmental processes [30–33]. piRNAs on the other hand have been primarily associated with processes protecting the genome against TE and viral activity, particularly in the germline [34–36]. piRNAs have been implicated in the maintenance and protection of germ cells in the zebrafish [37], chromatin repression of genomic regions with actively transposing elements in the house mouse [23], differential piRNA expression in sperm as a response to dietary changes in rats *Rattus norvegicus* [38], as well as the maintenance of metabolic homeostasis and normal lifespan in *Drosophila* [39]. In plants, on the other hand, non-genetic inheritance appears to be mediated by sRNA-dependent methylation pathways. In this pathway, the RNA Polymerase IV (Pol IV) generates precursor transcripts of sRNAs, which in turn target Pol V transcripts by sequence specificity to recruit specific domains of methyltransferase 2 [40]. Our model assumptions are generally based on the well-studied sRNA pathways in animals (e.g., miRNAs or piRNA regulated pathways). However, some general implications are directly applicable to other taxa and mechanisms, such as the feedback loops and possible resulting changes in transcription and amplification rates.

sRNA abundance is known to respond to changes in environmental conditions. In *Drosophila*, for example, changes in ambient temperature result in drastic but reversible changes in composition and abundance of ovarian miRNAs and piRNAs, with inversely correlated changes in their predicted targets [41,42]. Similarly, changes in temperature cause changes in miRNA expression in cotton *Gossypium sp*. [43] and rockcress *Arabidopsis* [44], and exposure to drought affects miRNA expression in tomato *Solanum lycopersicum* [45]. In the bacteria *Burkholderia thailandensis*, several environmental conditions such as pH, salt, antibiotics in addition to temperature have been shown to cause differential sRNA expression [46,47].

Recent evidence suggests that changes in sRNA expression levels may not only affect one generation but may be transferred across several subsequent generations [8,48]. In the nematode *Caenorhabditis elegans*, for example, viral infections led to a parental response that transformed viral double-stranded RNA into siRNAs, resulting in viral immunity. These siRNAs were transmitted from parents to offspring even in the absence of the initial stimulus and provided protection against the virus for up to three subsequent generations [49]. Further studies in *C*. *elegans* showed that starvation in one generation resulted in the transmission of sRNA-induced silencing of nutrition-related genes across three generations without further starvation [50], and nematodes exposed to increased temperature exhibited a differential sRNA-mediated gene-silencing response that was inherited for at least two generations after individuals were shifted back to lower temperature [51].

The magnitude of the sRNA response to environmental conditions and its transgenerational inheritance can depend on the duration of the stimulus (e.g., temperature stress) [48], indicating that sRNA levels may be responsive to the magnitude of environmental stress. The persistence of sRNAs across generations differs among sRNA families, but two characteristics facilitate sRNA inheritance: the ability to avoid removal by sRNA-degradation agents [52,53] and the ability to template their own synthesis via amplification cycles [54]. The sensitivity of sRNA production to environmental factors and the potential for transmission of sRNAs over several generations suggest that sRNAs can play an important role in adaptation.

The production of sRNA depends on two main processes, sRNA transcription from genomic DNA (generating primary sRNAs) and amplification from sRNA templates (generating secondary sRNAs), which can either directly act on their target transcripts or participate in a feed-forward mechanism of amplification to produce new secondary sRNAs [34]. This genome-independent amplification mechanism is particularly important for piRNAs and small-interfering RNAs (siRNAs) [34,55]. Several proteins, including members of the Argonaute protein family (e.g., Argo and Piwi) [56,57] and RNA-dependent RNA polymerases (RdRPs) [58,59], act as amplification agents by using secondary sRNA molecules as templates for the production of more sRNAs in amplification cycles known as the ping-pong cycle for piRNA and the RdRP-driven amplification cycle for siRNA.

sRNA levels, as well as the rate of amplification, can be transmitted from cell to cell. Within an individual generation, existing sRNAs are passed on to daughter cells, where they may be subsequently amplified [55,60,61]. Across generations, both sRNA molecules and the amplification agents (either the proteins themselves or the regulatory state of the corresponding genes) can be transmitted from parents to offspring [49,62–64], providing a mechanism that allows non-genetic inheritance of parental conditions [65,66].

**Fig 1:**
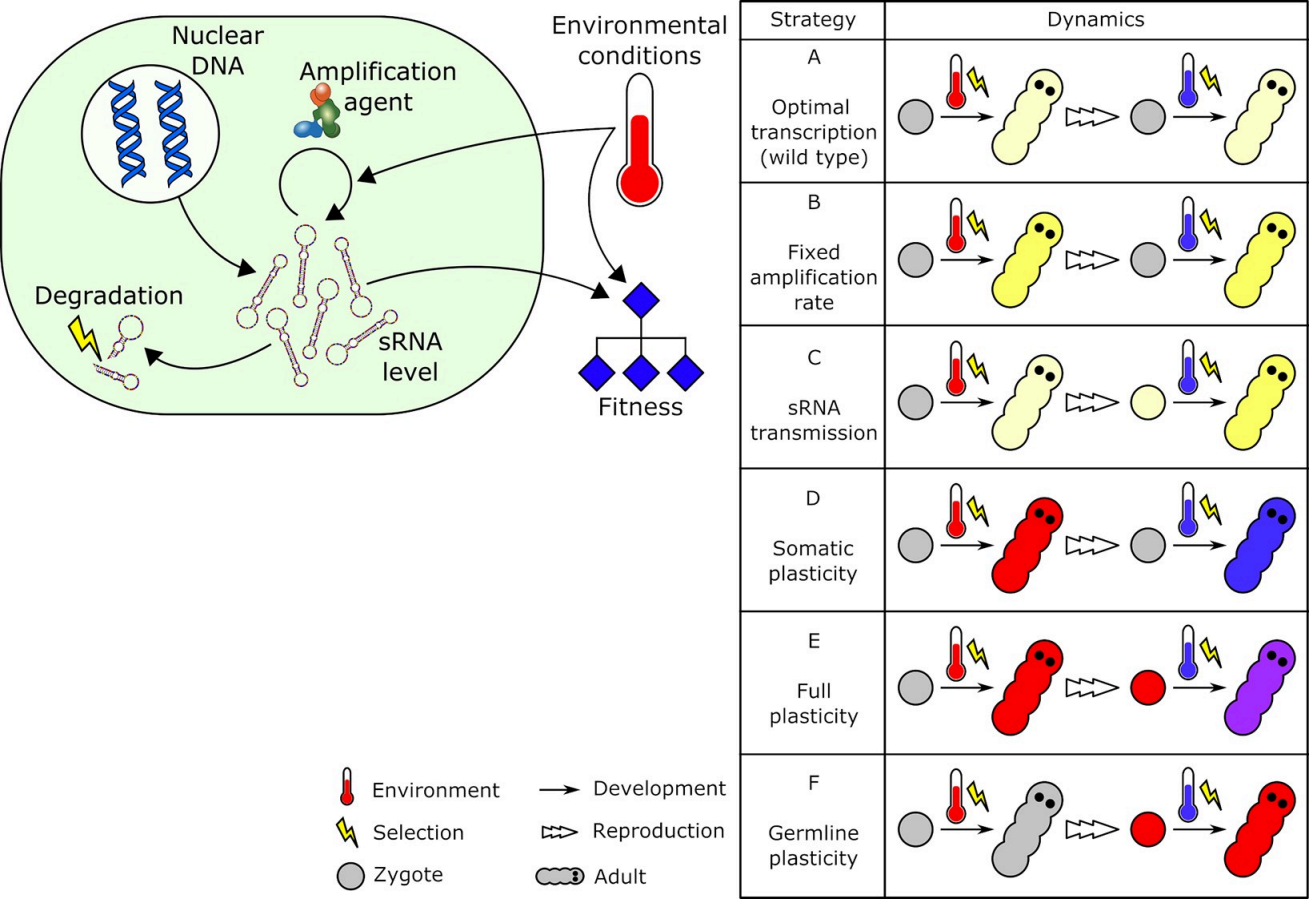
Model design. Left: sRNA production is a function of nuclear transcription, amplification and degradation processes. Fitness is a function of sRNA quantity and environmental conditions. Right: Strategies in the production and transmission of sRNA level or amplification rate that were explored in the simulations. Complete description in the main text.

Here, we develop a model to investigate how sRNA regulation and non-genetic inheritance evolve in a varying environment, thereby mediating plastic and transgenerational responses to the environment. We base many of our model assumptions on the well-studied sRNA mediated non-genetic inheritance mechanisms described in animals (such as piRNAs or miRNAs). However, many aspects of our model are more generally applicable. Our model allows both the primary production and secondary amplification of sRNAs, incorporates the effects of sRNA production on fitness as a function of the environment, considers the transfer of sRNA transcripts and amplification machinery across generations, and the potential costs of sRNA production. We explored under what environmental conditions different strategies of sRNA production and inheritance (as shown in **Fig 1**) are expected to invade a resident wildtype population with genetically fixed sRNA production and/or transfer rates. Our model and simulations provide a framework to study the evolution of sRNAs as important mediators of environmental conditions.

## Methods

We developed a model that consists of the following connected processes: (i) production, decay, and transmission of sRNA transcripts at the cellular level, (ii) sRNA inheritance across generations, (iii) plasticity in sRNA production through amplification, and (iv) a fitness function that depends on the costs and benefits of sRNA production in a given environment (**Fig 1**).

### sRNA production

sRNA production in our model is based on a feed-forward mechanism whereby the cellular level of sRNA (*n*, **Eq 1**) changes as a function of the transcription rate from nuclear DNA *μ*, the birth rate of sRNAs from existing template sRNAs, and the degradation rate *d*. Specifically, the total birth rate depends on the amplification rate *b* and on the number of existing templates *n*, rising linearly with *n* when templates are rare but approaching a maximum amplification rate of *m* when *n* is high:

$$\frac{dn}{dt}=\left(\frac{b}{1+b\frac{n}{m}}-d\right)n+\mu$$ Eq 1

**Eq 1** has two equilibria for *b*>0, only one of which is positive and is the biologically relevant equilibrium:

$$\hat{n}=\frac{b\;m+b\;\mu -d\;m+\sqrt{{\left(bm+b\mu -dm\right)}^{2}+4\;b\;d\;m\;\mu }}{2\;b\;d}$$ Eq 2

Time in **Eq 1** is measured in cell divisions. We assume that individuals replicate every *c* cell divisions and set *c* = 20, as a default value. This lies within the range of cell divisions per generation within the germline reported for different animal species [67]: 8.5 in *C*. *elegans*, 36 in *D*. *melanogaster*, and 200 in humans.

Additionally, sRNA transcripts can be directly transmitted across generations through the gametes of the parents, and the sRNA amplification rate can change in response to current or past environmental conditions (*ε*). We therefore distinguished between the amplification rate in the soma, b^*^, and the amplification rate in the germ line, *b*^*#*^.

Although sRNA transcription rates can also be plastic, here we focus on and isolate plasticity that comes solely through amplification of sRNA levels to understand how such plasticity in amplification rates evolve. This may be particularly relevant for sRNAs at sites that do not have environmentally sensitive transcription factors.

We then defined six possible strategies for the production, responsiveness and transmission of sRNAs and their amplification rate (**Fig 1**, right):

- Optimal transcription (wild type): Individuals have a fixed genetically determined sRNA production via transcription only, regardless of environmental conditions. There is no amplification (b^*^ = *b*^*#*^ = 0), plasticity or transgenerational transmission of sRNAs. Transcription rates are assumed to have evolved to their optimal levels.
- Fixed amplification rate: In addition to the optimal transcription rate in strategy A, strategy B individuals have a fixed genetically determined amplification rate (b^*^ = *b*^*#*^>0), without the ability to transmit sRNA levels or respond to environmental conditions.
- sRNA transmission: Similarly to strategy A, production of sRNA occurs via a fixed optimal transcription rate, but strategy C individuals can transmit sRNA levels to their offspring through their gametes.
- Somatic plasticity: Individuals have a plastic somatic amplification rate (b^*^) and a genetically fixed germline amplification rate (*b*^*#*^ = 0), allowing for a plastic response to changing environments in the parents without consequences for the next generation. In other words, in every generation, the somatic amplification rate b^*^ is reset to the inherited basal germline amplification rate *b*^*#*^ = 0 before it responds to environmental conditions.
- Full plasticity: Individuals have a plastic somatic amplification rate b^*^ and a plastic germline amplification rate *b*^*#*^, which change in response to current environmental conditions, and these changes are transmitted to the next generation. Changes are global, so both the soma and the germline are affected equally (b^*^ = *b*^*#*^).
- Germline plasticity: Individuals have a genetically fixed somatic amplification rate b^*^ and plastic germline amplification rate *b*^*#*^, which changes in response to current environmental conditions and is transmitted to the next generation and affects the somatic phenotype of the offspring. In this strategy, individuals do not directly benefit from having a plastic amplification rate but can potentially benefit their offspring by providing information about the parental environmental conditions.

Given a set of fixed biological parameter values (transcription rate, degradation rate and amplification activity), there is an optimal value of the amplification rate *b*, called *b*_*Wmax*,*g*_, that results in the highest fitness for a particular environmental condition *ε*. We assume that an individual can only adjust its amplification rate based on the information about current environmental conditions (no information about long-term environmental dynamics) and that plasticity has evolved to be adaptive, bringing the value of *b* closer to the optimal *b*_*Wmax*,*g*_ in that generation *g*. Plasticity is, however, limited and only allows the amplification rate to shift a fraction *P*_*b*_ of the way between the individual’s initial amplification rate *b* (inherited from the previous generation) and the optimum *b*_*Wmax*,*g*_. Somatic plasticity is absent in strategies A-C,F (*P*_*b*_ = 0) but present in strategies D,E (*P*_*b*_>0). Specifically, in generation *g*+1, the zygote inherits the amplification rate from the adult germline in the previous generation (*b*_*t*,*g*_^*#*^). Without somatic plasticity, the amplification rate *b*_*t*,*g*+1_^*^ throughout development remains equal to the level in the zygote:

$$b_{t,g+1}^{*}=b_{c,g}^{\#}\}\;\mathrm{Strategies}\;A‐C,\;F$$ Eq 3

With somatic plasticity, however, the amplification rate *b*_*t*,*g*+1_^*^ changes to:

$$b_{t,g+1}^{*}=b_{c,g}^{\#}+P_{b}\cdot (b_{W\max,g+1}-b_{c,g}^{\#})\cdot (1-e^{-a\cdot t})\}\;\mathrm{Strategies}\;D,\;E$$ Eq 4

where the term (1−*e*^−*a*∙*t*^) generates a delay in the plasticity response after exposure to a novel environment, and the plasticity level requires some time to reach the final level. This delay is defined by the constant *a*. We explored situations where plasticity is instantaneous (*a*→∞) and situations where plasticity is delayed (*a* = 0.15).

Transgenerational inheritance of the amplification rate, via transmission of templates, amplification agents, or epigenetic modification of those agents [68–70], is allowed in strategies E and F and is modeled via changes in the germline value of *b*^*#*^ from the adult parent (generation *g*) to adult offspring (generation *g*+1), depending on the environment experienced between these two stages:

$$b_{t,g+1}^{\#}=b_{c,g}^{\#}\}\;\mathrm{Strategies}\;A‐D$$ Eq 5

$$b_{t,g+1}^{\#}=b_{c,g}^{\#}+P_{b}\cdot (b_{W\max,g+1}-b_{c,g}^{\#})\cdot (1-e^{-a\cdot t})\}\;\mathrm{Strategies}\;E‐F$$ Eq 6

### sRNA transmission

Our model assumes that sRNA levels start very low in zygotes. However, sRNAs in the germline can be transmitted through the gametes from parents to their offspring [49,63,64]. Because of that, our assumption of low zygotic sRNA level can be relaxed by allowing maternal inheritance of sRNA transcripts. This transmission, which distinguishes strategy C from the wildtype strategy A, provides a second opportunity for transgenerational inheritance of sRNAs, beyond the inheritance of plastic changes in amplification rate. To model the transmission of sRNAs from parents to offspring, we assume maternal inheritance and vary the fraction *r*_*germ*_ of sRNAs that could be passed from mother to zygote. Specifically, given the quantity of sRNAs in adult female germ cells *n*_*final*_, the amount inherited in the zygote *n*_*initial*_ in the next generation is set to *n*_*initial*,*g+1*_ = *r*_*germ*_ *n*_*final*,*g*_. We assume that *n*_*final*_ results from the production of sRNAs throughout development according to **Eq 1** and is the same in the germline and in somatic cells. In addition to being a characteristic of Strategy C, we briefly explore the evolution of the transmission ratio *r*_*germ*_ for strategies D-F.

### Environmental conditions

sRNA production is known to respond to a variety of environmental variables [41–44,46,47,50]. To model a response to the environment, we include a continuous variable *ε* measuring the impact of the environment on individual fitness 0≤*ε*≤1, where detrimental conditions (*ε*>0) are deviations from the optimal environmental condition (*ε* = 0). For ease of reference, *ε* will represent a measure of how stressful the environment is to the organism. In benign environments (*ε* = 0), maximum fitness is achieved even when producing low to no amounts of the particular sRNA being modeled. In stressful environments, maximum fitness is achieved only by producing high amounts of this sRNA.

Transgenerational inheritance is predicted to be favorable when environmental conditions are more similar between parents and offspring than between more distant points in time (i.e., positively autocorrelated) [71]. In order to explore the impact of environmental autocorrelations, we hold the fraction of time that the environment was stressful (*ε* = 0.9) or benign (*ε* = 0.1) constant at 50% each ($\bar{\varepsilon }$ = 0.5). Thus, organisms always face the same array of environments, just in a different order over time. Specifically, we use the probability that the environment is the same for parents and offspring, $p_{\varepsilon }=1-\frac{k}{G-1}$, as a measure of the environmental similarity of parents and offspring, where *k* is the total number of changes in environmental conditions (changes from *ε* = 0.1 to *ε* = 0.9 or vice-versa) and *G* is the total number of individual generations in each environmental scenario cycle (*G* = 20). High environmental similarity (high *p*_*ε*_) indicates that the environment switches rarely (positively autocorrelated), while a value of zero indicates switching every generation (negatively autocorrelated). Specifically, we consider environmental scenarios with *p*_*ε*_∈{0.11, 0.53, 0.89} (see examples in **S1 Fig**). For tractability, we assume that a given environmental scenario repeats every *G* = 20 individual generations, so that we could determine the long-term fitness of each strategy.

### Fitness

We model fitness in a manner that allows for costs of plasticity and sRNA production (for example, the costs of activating a molecular pathway needed to deal with specific environments, although this is not explicitly modelled here) as well as benefits of matching the environment across the lifespan of the organism. At each time step *t* during development, individual fitness is given by a continuous function *W*(*n*_*t*_, *ε*_*g*_,*P*_*b*_) of the quantity of sRNA (*n*_*t*_), the current environmental condition (*ε*_*g*_) in generation *g*, and the degree of plasticity in amplification rate (*P*_*b*_). For this fitness function, we used:

$$W(n_{t},\varepsilon_{g},P_{b})=\frac{1}{1+C_{n}n_{t}+C_{b}P_{b}}exp\left[-\left(\beta +\alpha \varepsilon_{g}\right){\left(\frac{e^{n_{t}}-1}{e^{n_{t}}+e^{h}-2}-\varepsilon_{g}\right)}^{2}\right]$$ Eq 7

where the first term represents the cost (*C*_*n*_) associated with sRNA production (*n*) plus the cost associated with the plastic response in amplification rate *b* (*C*_*b*_). The second term of the fitness function is the benefit of matching sRNA production to environmental conditions. Specifically, fitness is assumed to decline exponentially with the degree of environmental stress (given by *β*+*α ε*_*g*_), but this decline is ameliorated by a match between the sRNA “phenotype” and the environment, *ε*_*g*_. The phenotype is a logistic-shaped function of the quantity of sRNA, *n*_*t*_, equaling 0 in the absence of sRNA and rising with large amounts of sRNA to 1, representing the best match to the most stressful environment (*ε*_*g*_ = 1). The parameter *h* represents the amount of sRNA required to match an intermediate environmental stress of *ε*_*g*_ = 0.5.

The form of **Eq 7** allows asymmetries in the fitness effects of sRNA production in stressful and benign environments (**S2 Fig**). Specifically, the production of sRNAs in benign conditions is assumed to have a milder fitness effect than the benefits of production under stressful conditions. A plastic amplification rate (*P*_*b*_>0) has the benefit of bringing sRNA amounts closer to the optimum under different environmental conditions and can strongly increase fitness, but this plasticity in amplification rate comes at a cost (*C*_*b*_ *P*_*b*_).

We assume a simple genetic model where each strategy can be encoded by a mutation at a single gene, in which case a mutant strategy will spread within a wildtype population if it has a higher geometric mean fitness throughout development (*W*_*LIFE*,*g*_) and over the set of environments encountered (*W*_*GEO*_), where:

$$W_{LIFE,g}={\left(\prod_{t=0}^{c}W(n_{t},\varepsilon_{g},P_{b})\right)}^{\frac{1}{c+1}}$$ Eq 8A

$$W_{GEO}={\left(\prod_{g=1}^{G}W_{LIFE,g}\right)}^{\frac{1}{G}}$$ Eq 8B

With sRNA transmission across generations (*r*_*germ*_>0), the amount of sRNAs in the zygote, *n*_*initial*_, depends on ancestral production of sRNA and varies across the repeated *G* = 20 periods; we thus calculated the geometric mean fitness after allowing *n*_*initial*_ at the beginning of a period to reach a stable value.

We next determine the environmental conditions under which mutant strategies (strategies B-F; superscript "+") can invade a resident wildtype population (strategy A; superscript "–"), with parameters summarized in **Table 1**. To assess the strength of selection, on average, in favor of a mutant strategy, we use the selection coefficient *s*^*+*^ calculated from the relative geometric mean fitness of the mutant:

$$s^{+}=\frac{W_{GEO}^{+}}{W_{GEO}^{-}}-1$$ Eq 9

Because we are particularly interested in cases where selection is strong, we illustrate where selection on the mutant becomes weaker in magnitude than |*s*^*+*^| = 0.001, as drift will then dominate selection except in populations of moderately large size (*N*>1000) [72].

**Table 1:** Parameter values used for each strategy in the invasion analyses. S and G indicate plasticity in the soma and germline, respectively. Values in bold indicate mutant values.

Strategy	*b*	*d*	*m*	*μ*	*r*_*germ*_	*P*_*b*_
**A** (optimal transcription)	0	0.1	5.0	6.798	0	0
**B** (fixed amplification rate)	**>0**	0.1	5.0	6.798	0, **>0**	0
**C** (sRNA transmission)	0	0.1	5.0	6.798	**>0**	0
**D** (somatic plasticity)	**>0**	0.1	5.0	6.798	0**, >0**	0 (**G**), >0 (**S**)
**E** (full plasticity)	**>0**	0.1	5.0	6.798	0**, >0**	>0 (**S**,**G**)
**F** (germline plasticity)	**>0**	0.1	5.0	6.798	0**, >0**	0 (**S**), >0 (**G**)

Although there is some evidence that the mRNA degradation rate is not constant and changes according to mRNA production [73], we believe that a constant intrinsic degradation rate is a reasonable simplification of the process and assume that the sRNA degradation rate *d* is an intrinsic stability property of the sRNA molecule under focus and set it to *d*^–^ = *d*^*+*^ = 0.1. Similarly, the maximum amplification rate, *m*, is set to *m*^–^ = *m*^*+*^ = 5.0. We further assume that the rate of sRNA transcription from DNA has evolved to maximize the geometric mean fitness of strategy A in the absence of plasticity or transgenerational inheritance, setting this optimum parameter value as the default (*μ*^−^ = *μ*^+^ = 6.798 for the environmental conditions used in our simulations with $\bar{\varepsilon }$ = 0.5 and *ε*∈{0.1, 0.9}). For strategies allowing sRNA amplification (B, D-F), we consider all positive values of *b*, assuming the value yielding the highest geometric mean fitness would eventually arise (*b*_*Wmax*,*g*_) and using this value to assess whether the mutant strategy could invade.

Table 2 contains a brief description of all the parameters used in the model and simulations. All analyses and simulations were performed in Wolfram *Mathematica* 11, version 11.0.1.0. A *Mathematica* notebook containing all of the analyses is available within the Supporting Information files (**S1 Appendix**).

**Table 2:** Parameters used in the model and simulations in alphabetical order and latin followed by greek alphabet.

Parameter	Definition
*-*	Superscript indicating wildtype parameters
*+*	Superscript indicating mutant parameters
*a*	Magnitude of the delay in plasticity
*b*	sRNA amplification rate
b^*^	Somatic sRNA amplification rate
*b*^*#*^	Germline sRNA amplification rate
*b*_*Wmax*_	Optimal amplification rate in given environment (*ε*)
*c*	Number of cell divisions per generation
*C*_*n*_	Cost of sRNA production
*C*_*b*_	Cost of change in sRNA amplification rate *b*
*d*	sRNA degradation rate
*g*	Current generation
*G*	Number of generations over which environmental conditions repeat themselves
*k*	Total number of changes in environmental conditions
*m*	Maximum sRNA amplification rate
*n*	Amount of sRNAs per cell
*n*_*initial*_	Amount of sRNAs inherited into the zygote
*n*_*final*_	Amount of sRNAs in female adult germ cells
*P*_*b*_	Plasticity level
*r*_*germ*_	sRNA fraction transferred from mother to zygote
*s*	Selection coefficient favoring mutant
*t*	Developmental time (in cell divisions)
*W*_*LIFE*_	Geometric mean fitness during development
*W*_*GEO*_	Geometric mean fitness across all environmental conditions (*ε*) across generations
*α*	Fitness function shape parameter
*β*	Fitness function shape parameter
*ε*	Environmental condition
*p*_*ε*_	Environmental similarity
*μ*	Transcription rate

## Results

**Fig 2:**
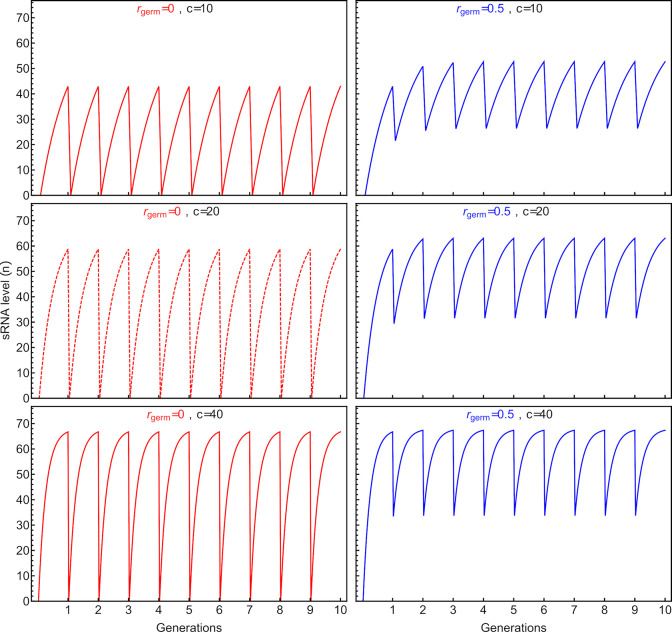
Dynamics of sRNA within and between generations, for different transmission ratios (*r*_*germ*_ = 0 left; *r*_*germ*_ = 0.5 right) and different numbers of cell divisions per generation (*c*; top to bottom). Red dashed curves represent the default dynamics of sRNA production (as used in strategy A). The middle blue curve represents an example of strategy C (*r*_*germ*_ = 0.5). Parameter values used in the example: *b* = 0, *d* = 0.1, *m* = 5.0, *μ* = 6.798. All subsequent simulations use *c* = 20 (middle panels).

During individual development, the production of sRNA rises, approaching the steady state value, $\hat{n}$ (**Eq 2**), at a rate that depends on the sRNA strategy (**Table 1**) and the parameters responsible for sRNA production and maintenance (*b*, *d*, *m* and *μ*; **S3 Fig**). The level of sRNA reached in adulthood thus depends on the number of cell divisions from the start to the end of the generation, *c*, as well as the initial fraction of sRNA inherited by the zygote, *r*_*germ*_. The transmission ratio (*r*_*germ*_) determines how much sRNA levels are reset from generation to generation and hence the degree of sRNA oscillations witnessed across individual generations (**Fig 2**).

Without plasticity or non-genetic inheritance (*b* = 0, *r*_*germ*_ = 0, and *P*_*b*_ = 0), the fitness of strategy A is maximized at a transcription rate of *μ* = 6.798 (**S4 Fig**), regardless of the order of environments encountered. The adult level of sRNA then reached ~58.8 for strategy A (solving **Eq 1** at *c* = 20), which lies between the optimum adult level of 3.2 if the environment were always benign (*ε* = 0.1) and 65.3 if always stressful (*ε* = 0.9). This adult level is much higher than one would expect from the fitness landscape at any point within a generation (*W*(*n*_*t*_,*ε*_*g*_,*P*_*b*_); **S2 Fig**). For example, at time *t* during development, the optimum sRNA level is only *n* = 2.85 for *ε* = 0.1 and *n* = 7.19 for *ε* = 0.9. The optimal transcription rate causes the adult sRNA to overshoot what is needed at that life stage in order to ensure enough sRNA earlier in life. That is, as assumed in **Eq 1**, excess sRNA production is more beneficial than insufficient production.

### Evolution of mechanisms for sRNA amplification and inheritance

**Fig 3:**
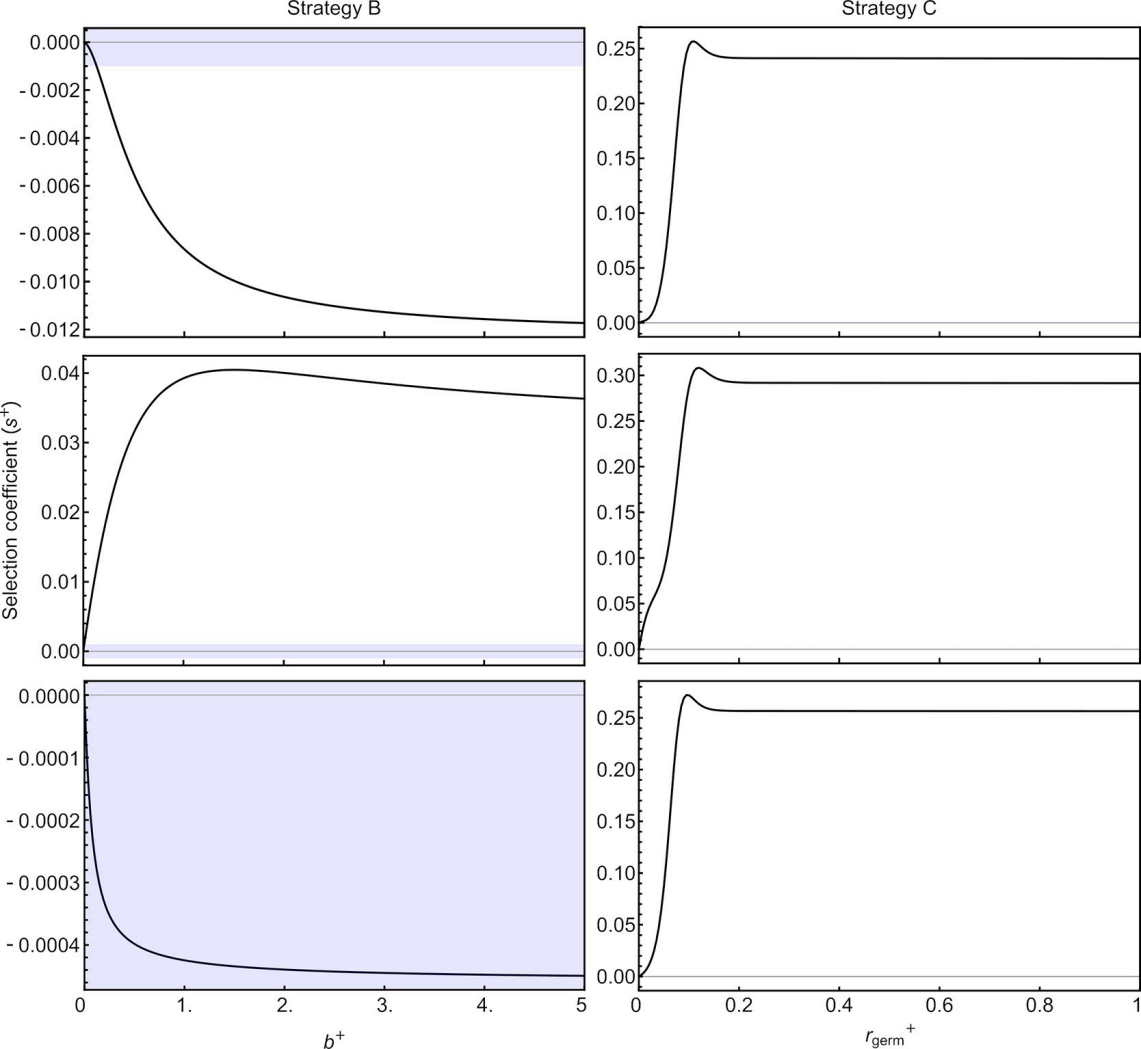
Selection for amplifying sRNA at a fixed rate (*b*^*+*^>0; strategy B in left panels) and selection for cross-generational transmission of sRNA transcripts (*r+germ*>0; strategy C in right panels) in a resident population of strategy A (*b*^*-*^ = 0 and *r-germ* = 0). Upper panels show fitness levels under default settings (transcription rate optimal for strategy A), middle panels show fitness levels under settings slowed by a factor 0.75, and bottom panels show fitness levels under settings sped up by a factor 2.0. Blue shaded regions denote weak selection coefficients (|*s*^*+*^|≤0.001).

Mutants capable of amplifying sRNA production via a fixed amplification rate (*b*^*+*^>0, strategy B) are unable to invade a resident population of strategy A (i.e., *s*^*+*^<0) under the environmental conditions to which the resident population is already optimally adapted (*μ* = 6.798). However, mutants capable of transmitting sRNA across generations (*r*^*+*^_*germ*_>0, strategy C) have a selective advantage relative to the resident population of strategy A (**Fig 3**, upper panels). While increasing amplification allows sRNA levels to accumulate faster (**S3 Fig**), only the transmission of sRNA transcripts from parents to offspring (*r*^*+*^_*germ*_>0, strategy C) allows high levels of sRNA to be reached early in life (**Fig 3**, upper panels), buffering juveniles from stressful environments. As strategies A-C do not have a plastic response to the environment, the results are insensitive to the order of environments encountered (*p*_*ε*_).

Allowing pleiotropy between sRNA amplification and transmission (combining strategies B+C), we find that the combination of sRNA amplification (*b*^*+*^>0) and transmission (*r*^*+*^_*germ*_>0) can be as advantageous as transmission alone, and sometimes more so (**S5 Fig**). This is due to the increased fitness benefit provided to the offspring during early development through the inheritance of an increased amount of sRNAs in stressful generations.

In the above comparisons, we assume that strategy A has an sRNA transcription rate that is the best possible fixed strategy for the environmental conditions experienced. This explains why strategy B on its own never helps (**Fig 3**, top left panel), because increasing sRNA levels by amplification beyond the already optimal level produced via transcription does not increase fitness. However, natural systems may be constrained from reaching the optimal sRNA level within an individual generation by transcription alone or overall dynamics may happen at a faster pace. To explore such constraints, we investigate a case where the dynamics are slowed by a factor 0.75 (multiplying *b*, *d*, *m*, and *μ* by 0.75), in which case the same steady state level of sRNA would be reached but over a longer period of time (**S3 Fig**), and a case where the dynamics are sped up by a factor 2.0 (multiplying *b*, *d*, *m*, and *μ* by 2.0), in which case the same steady state level of sRNA would be reached but over a shorter period of time (**S3 Fig**). With slower dynamics, sRNA levels do not reach the optimal fixed level within one generation. In this case, we find an advantage to amplification of sRNA, and sRNA transmission becomes even more beneficial (**Fig 3**, middle panels). On the other hand, with faster dynamics, sRNA amplification is slightly detrimental, while sRNA transmission continues to be beneficial (**Fig 3**, bottom panels).

### Evolution of environmentally responsive sRNA amplification and inheritance

We next explore plastic sRNA amplification (strategies D-F) and determine whether a mutant capable of responding to environmental conditions (*P*_*b*_^*+*^>0) could invade a resident population of non-plastic individuals (strategy A; *b* = 0 and *P*_*b*_^*-*^ = 0). We consider various degrees of plasticity, from weakly responsive (*P*_*b*_^*+*^ near 0) to perfectly responsive (*P*_*b*_^*+*^ near 1), considering only adaptively plastic strategies that increase amplification in stressful environments (*P*_*b*_^*+*^>0, see **Eqs 3–6**). We also consider instant plasticity (*a*→∞), where individuals adjust their amplification rate immediately upon contact with environmental conditions, and delayed plasticity (*a* = 0.15), where the amplification rate changes gradually throughout development towards its final value.

**Fig 4:**
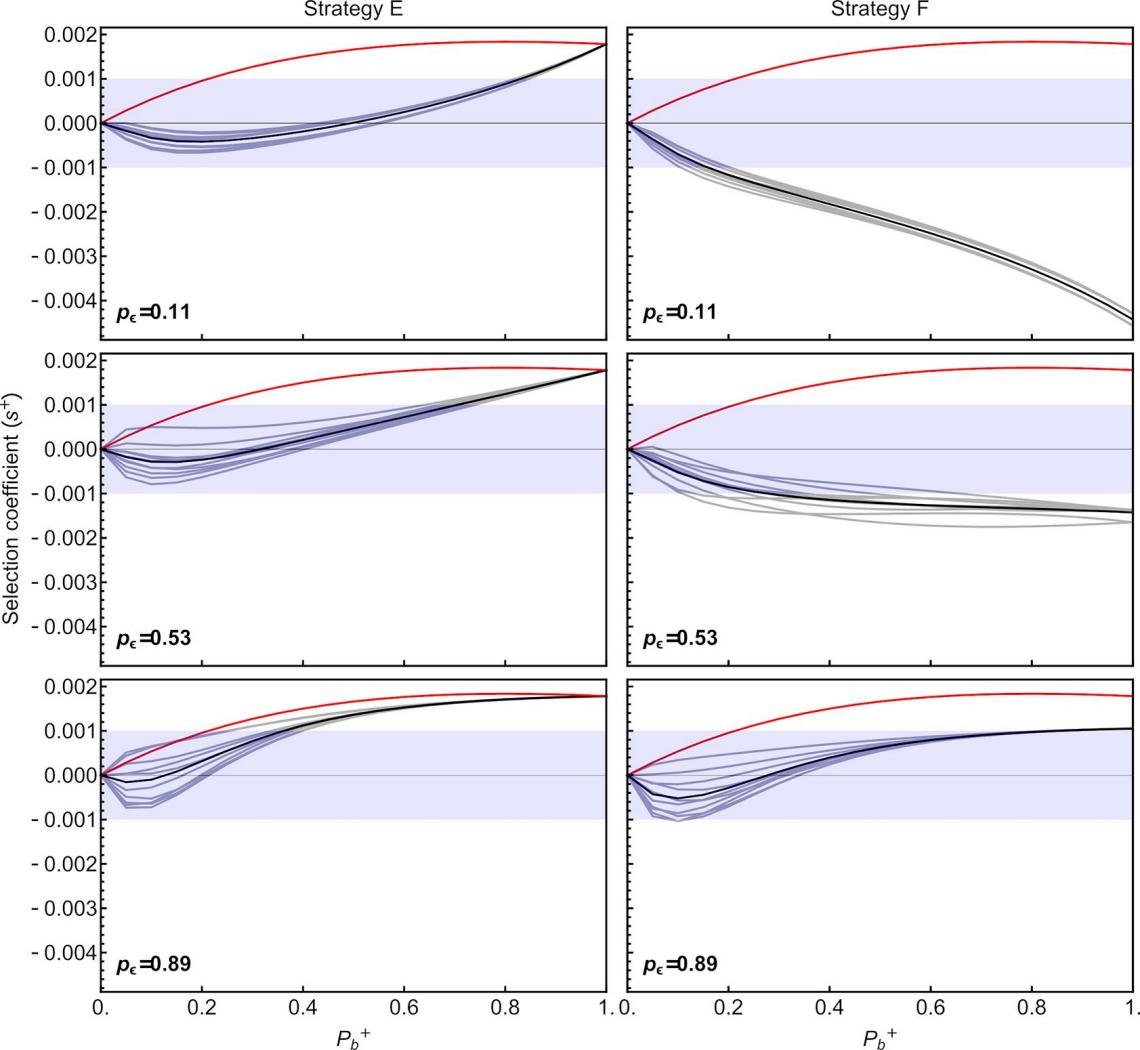
Selection on strategies that instantly amplify sRNA in response to environmental conditions (strategies D-F). Red curves represent somatic plasticity (strategy D). For full plasticity (strategy E, left) or germline-only plasticity (strategy F, right), the black curves represent the mean selection coefficient across different realizations (gray curves) of environmental scenarios. Germline plasticity is most favored when environmental fluctuations are positively autocorrelated over time, such that parents and offspring are likely to encounter the same environment (*p*_*ε*_ high). Blue shaded regions denote weak selection coefficients (|*s*^*+*^|≤0.001).

We first consider mutants only capable of instant somatic changes in amplification rate based on the current environment (strategy D; **S6 Fig**). Because such mutants increase the production of sRNA specifically when the environment is stressful, strategy D mutants are selectively advantageous (*s*^*+*^>0) compared to the resident strategy A, for the parameters considered (**Fig 4**; red curves). Because the response is restricted to the soma, the similarity in environments experienced by parents and offspring does not affect selection for the plastic strategy D (red curves are insensitive to changes in *p*_*ε*_ from top to bottom panels in **Fig 4**). The selective benefit of strategy D is strongest at intermediate degrees of responsiveness to the environment (highest selective advantage when *P*_*b*_^*+*^ = 0.8), reflecting a balance between the benefits of having some plasticity and the costs of responding precisely.

Selection for plastic sRNA amplification depends strongly on the cost of plasticity. Increasing this cost from *C*_*b*_ = 50*C*_*n*_ to *C*_*b*_ = 500*C*_*n*_ largely eliminated the benefits of strategy D under the default parameters (**S7 Fig**). Essentially, because we allow strategy A to evolve first towards its optimum in the absence of plasticity, plastic sRNA amplification is favored only if it involves sufficiently low costs.

When plastic changes in the amplification rate can be transmitted to offspring, the selective advantage of mutants depends on the similarity of environments encountered by parents and offspring (*p*_*ε*_) and whether plastic sRNA amplification affects both the soma and germline (strategy E) or only the germline (strategy F) as illustrated in **Fig 4**. With transgenerational inheritance (strategies E and F), the exact order in which environments are encountered now influences the strength of selection (gray lines in **Fig 4**), even holding constant both the array of environments ($\bar{\varepsilon }$ = 0.5 with *ε*∈{0.1, 0.9}) and the level of environmental similarity (*p*_*ε*_). Again, only when costs of plasticity are sufficiently low (small *C*_*b*_ *P*_*b*_) does selection favor plastic strategies E and F over the wildtype (compare **Fig 4** and **S7 Fig**).

Having both a plastic somatic and germline response (strategy E) is particularly favored when the environment remains the same for several generations (high *p*_*ε*_). The majority of this advantage comes from somatic plasticity, as strategy D (somatic plasticity only) remains fitter across the range of conditions explored. Indeed, a plastic response restricted to the germline (strategy F) is never as strongly advantageous, because the restriction of amplification changes to the germline provides a fitness benefit only when the current and parental environments are both stressful. As a consequence, strategy F is favored over the fixed wildtype strategy A only when the environment remains similar between parents and offspring (*p*_*ε*_ = 0.89).

Again, we hypothesize that plasticity would be more favorable if sRNA levels are constrained from reaching the optimum for strategy A. When we slow down the dynamics (multiplying the within-generation parameters *b*, *d*, *m*, and *μ* by 0.75), all plastic strategies (D-F) become favored over the wildtype strategy A (**S8 Fig**). Furthermore, transgenerational inheritance is much more commonly favored. Specifically, strategies with germline plasticity (E and F) increase fitness more than strategy D with only somatic plasticity (**S8 Fig**), as long as plasticity and its associated costs are low (small *C*_*b*_ *P*_*b*_). When sRNA production dynamics happen faster (twice the default speed), somatic and/or germline plasticity (strategies D-F) are no longer advantageous (not shown). Increasing the speed of the developmental dynamics such that optimal sRNA levels are reached very early during development eliminates the benefit of plasticity at least for the parameters investigated here where plasticity is costly and not sufficiently beneficial when sRNA levels rise rapidly during development.

**Fig 5:**
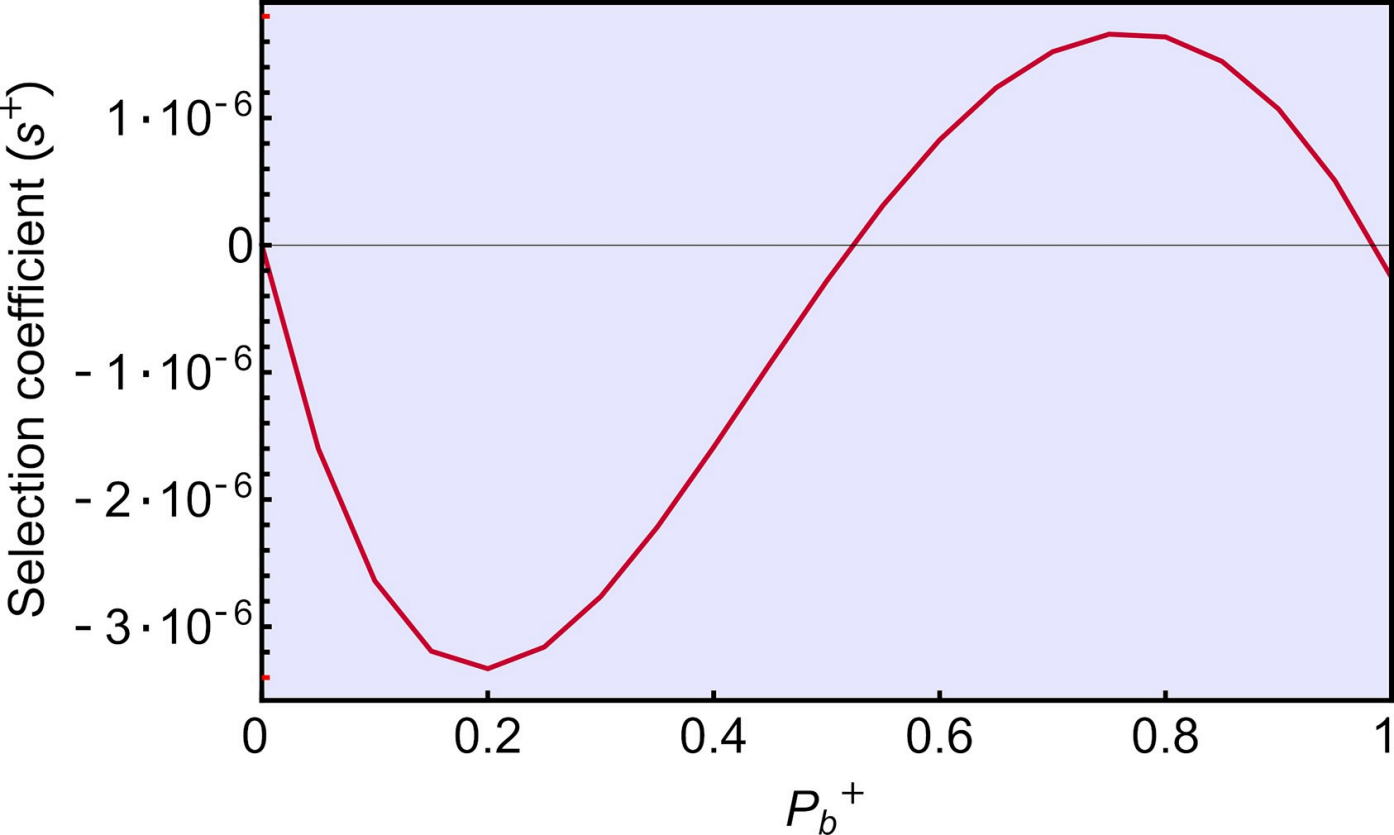
Selection on somatic plasticity (strategy D) that responds to environmental conditions by gradually increasing sRNA amplification rates during development (*a* = 0.15). The blue shaded region denotes weak selection coefficients (|*s*^*+*^|≤0.001).

**Fig 6:**
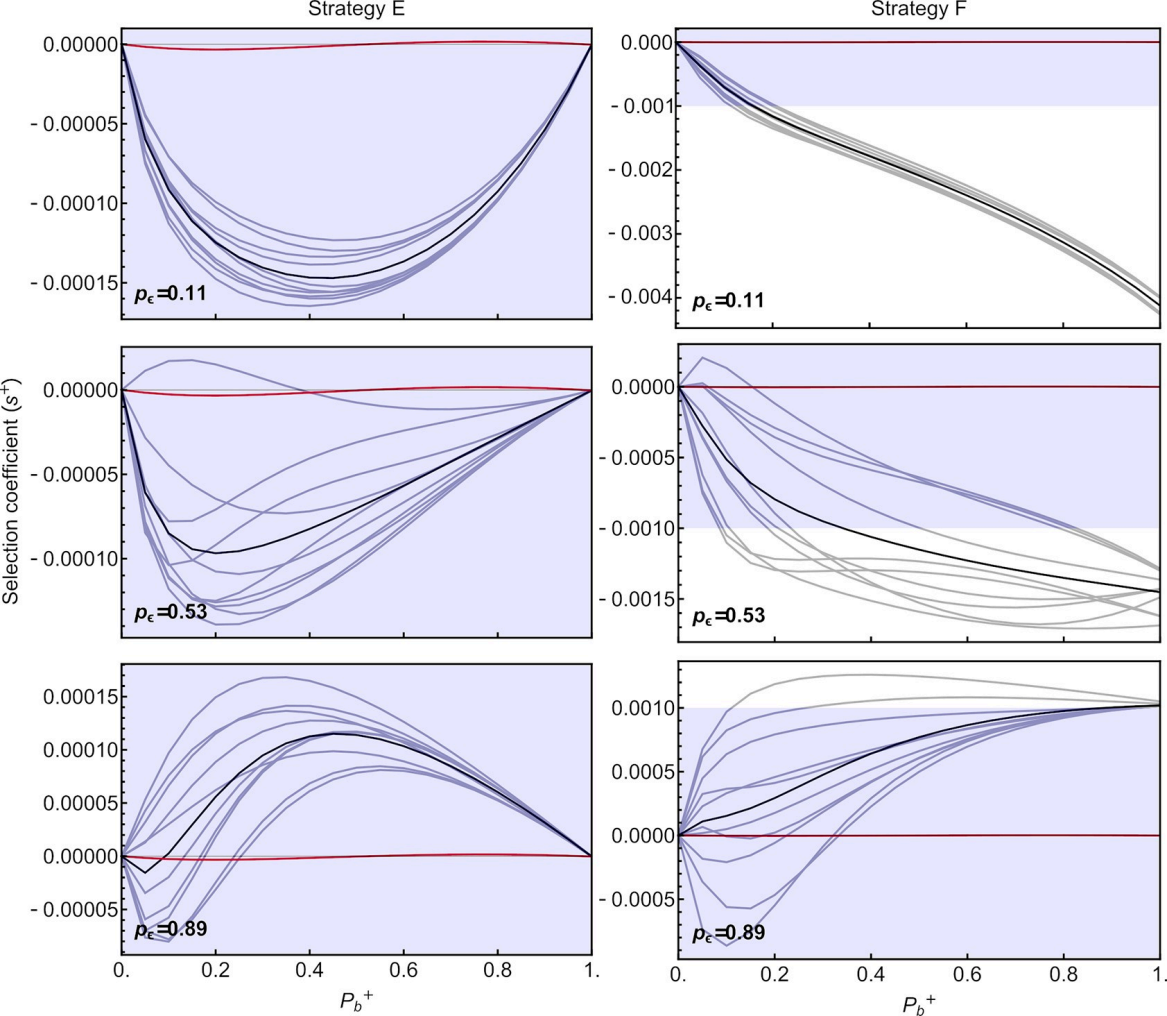
Selection on strategies that respond to environmental conditions by gradually increasing sRNA amplification rates during development (*a* = 0.15). Selection on somatic plasticity (strategy D; red curves as in **Fig 5**) is very weak and hardly visible, relative to selection coefficients on germline plasticity (strategies E-F, black curves). See **Fig 4** for additional details.

Next, we explore a more gradual plastic response to the environment during development (*a* = 0.15), instead of instantaneous. In such cases, somatic plasticity alone (strategy D) is only slightly beneficial when plasticity is strong (high *P*_*b*_, **Fig 5**). Because of the delay in the plastic response, plasticity makes less of a different during the early stages of development, unless it has a major effect on amplification. Germline plasticity, either in addition to somatic plasticity (strategy E) or alone (strategy F), is only beneficial (and more so than somatic plasticity alone) when the parental and offspring environments are highly similar (*p*_*ε*_ = 0.89; **Fig 6**). This benefit of germline plasticity comes in the form of a head start in amplifying sRNAs during early development, allowing strategies with germline plasticiy to approach the optimal sRNA levels for current environmental conditions sooner.

Although not included in our model, plastic transcription rates (with or without transgenerational inheritance) are also possible but they are limited to one target (the DNA sequence encoding the sRNA), whereas sRNA amplification allows faster (exponential) growth in transcripts by replicating the larger number of existing sRNAs. Because of that, we expect that, in the absence of amplification, a plastic transcription rate would evolve similarly to the plastic amplification rates explored in our model, but towards greater values than amplification rates due to the non-exponential sRNA production by transcription, that is, higher transcription rates compensate for the absence of (exponential) amplification rates. More complicated models allowing plasticity in both transcription and sRNA amplification rates would be worth exploring to determine the conditions under which each is more likely to evolve.

Finally, we explore the selective advantage of mutations altering the germline transmission of sRNA in the context of instantaneous plastic sRNA amplification by determining the full two-dimensional fitness landscape with respect to both plasticity in amplification rate (*P*_*b*_^*+*^>0) and sRNA transmission (*r*^*+*^_*germ*_>0). For ease of presentation, we randomly selected one environmental scenario with low environmental similarity (*p*_*ε*_ = 0.11) and one scenario with high environmental similarity (*p*_*ε*_ = 0.89) to illustrate the fitness surface (**S9 Fig**). For these parameters, the highest fitness strategies always involve inheritance of sRNA transcripts and never involve plasticity (the fitness maxima illustrated by black points in **S9 Fig** occur at *r*^*+*^_*germ*_ = 0.11 and *P*_*b*_^*+*^ = 0). Thus, for the parameters considered, transgenerational inheritance (*r*^*+*^_*germ*_>0) evolves primarily to ensure sRNA levels are sufficient early in life. Additional plastic amplificiation is then not favored, at least not when the resident transcribes sRNA at the fixed rate *μ*^−^ = 6.798, which together with cross-generational transmission provides sufficient sRNA levels regardless of the environment.

## Discussion

Modeling the dynamics of sRNA that buffer the organism against stressful environments, we find that mechanisms that allow sRNA levels to be amplified somatically under stressful conditions are favored unless the costs of plasticity exceed the benefits (strategy D; **Fig 4**). By contrast, germline transmission of sRNA amplification rates across generations via non-genetic inheritance of sRNA templates, amplification agents, or epigenetic up-regulation of those agents (strategies E or F) is selectively favored over somatic plasticity (strategy D) only if sRNA levels remain below the optimal levels throughout development due to downregulation of transcription (**Fig 5**). However, transmitting sRNA transcripts directly to offspring (strategy C) provides fitness benefits (**Figs 3** and **S8**) over the wildtype (strategy A) even when the optimal sRNA level could be reached in the adult. Transmission of transcripts provides immediate fitness benefits to the offspring during early development, when the level of transcribed sRNAs is still low.

Previous theoretical studies have found similar results regarding the benefits of non-genetic inheritance (e.g., maternal effects) under various environmental conditions. It has been shown that phenotypic memory in the form of a positive correlation between parental and offspring phenotype is favored when environmental conditions are relatively stable [71]. As the rate of change of environmental conditions increase, that correlation decreases and selection favors less faithful transmission of phenotypes. When maternal effects are considered from a multivariate perspective, selection favors a positive correlation between the multivariate phenotypes of the offspring and the mother when the rate of environmental fluctuations is low, but a negative correlation in rapidly changing environments [74]. Furthermore, informative maternal effects were shown to be beneficial when juvenile cues are inaccurate, transmission of maternal cues are accurate, and the environment is highly stable [75]. Although the transmission of phenotypes from parent to offspring may be beneficial under certain environmental conditions (mentioned above), parents and offspring may have conflicting interests that can affect the evolution of information transfer from parents to offspring. A different study explored the effect of such parent-offspring conflicts and showed that, in many cases, it causes a partial or complete breakdown of informative maternal effects, which may explain the apparent weakness of transgenerational plasticity in nature [76].

When fitness is affected by environmental conditions, different systems of phenotypic determination can evolve depending on the accuracy of genetic versus environmental cues. When genetic cues are accurate and environmental cues are inaccurate, phenotypic determination based on genetic polymorphism is likely to evolve. However, when genetic cues are inaccurate and environmental cues are accurate, phenotypic plasticity is more likely to evolve [77–79]. These outcomes have important consequences for the interpretation of our results, in particular for strategies E and F, where transgenerational plasticity is present. In highly autocorrelated environments (high *p*_*ε*_), parents employing strategies E and F have an accurate environmental cue and can benefit their offspring by transmitting their sRNA amplification rate to gametes, which is similar to results from previous models on maternal effects without the amplification dynamics considered here [80]. With weakly or negatively autocorrelated environments, however, the sRNA amplification rate transmitted by the parents is frequently detrimental to the offspring, thereby favoring somatic plasticity without transmission (strategy D over strategies E and F), unless transgenerational inheritance is needed to reach high sRNA levels (compare **Fig 4 and S8 Fig** for fast and slow dynamics, respectively). Additionally, previous results have concluded that maternal effects are more strongly favored when plasticity is limited [80], which is consistent with our results that non-genetic inheritance of sRNA amplification rates (strategy F) is typically not favored if a highly plastic strategy is available (strategy D). However, we also show that plasticity (somatic and/or germline) loses its benefit when optimal sRNA levels are reached very early during development.

Unlike previous theoretical studies, however, our study provides solid, clear predictions for the well-known molecular mechanism of sRNA-mediated phenotypic plasticity, sRNA amplification, and non-genetic inheritance. By exploring a range of possible strategies (**Fig 1**), we can predict which type of molecular mechanisms might evolve under different environmental scenarios:

- sRNA amplification (strategy B) evolves when optimal sRNA levels cannot be reached via transcription within a single generation (**Fig 3,** middle left).
- Transmission of sRNA transcripts (strategy C) from parents to offspring evolves because of the fitness benefit provided to the offspring early in development, while sRNA levels from transcription remain low (**Fig 3**, right panels).
- Somatic plasticity in sRNA amplification (strategy D) is fittest when the benefits of increasing amplification in response to stressful environments outweigh the fitness costs (**Fig 4**, red curves).
- When the plastic response is gradual, somatic plasticity (strategy D) is only beneficial when plasticity is strong, so that the additional production of sRNA via amplification outweights the cost of plasticity during early development (**Fig 5**).
- With gradual plastic responses, germline plasticity is only beneficial when parental and offspring environments are very likely to be similar (**Fig 6**).
- With instant plasticity responses, germline plasticity in sRNA amplification (strategy E or F) is fittest only when constraints prevent sRNA levels from reaching high enough levels from transcription alone (**S8 Fig**).

Our model thus provides a valuable reference for future empirical studies testing the effect of sRNA-related mutations and the effect of environmental stress on such mutants. The hypotheses proposed here can be tested with, for example, the use of molecular technology (e.g., CRISPR-Cas9) to impair the regulatory machinery responsible for amplification, transmission or responsiveness of a target sRNA. This can be followed by fitness assays that test the effect of environmental similarity on survival and reproduction of impaired mutants.

The predicted evolutionary response of sRNA production and transmission to changing environments obtained from our model is in line with recent empirical observations in the nematode *C*. *remanei* [81]. Nematodes were exposed to four different environments including stable at a control (20°C) or high temperature (25°C), or in slowly or rapidly changing environments. Lines exposed to stable or slowly changing environments showed a strong positive maternal effect on reproductive output resulting in increased offspring production, whereas lines maintained in a rapidly changing environment showed a reduced maternal effect on offspring production. This finding is consistent with our results and others [71,75,82] that the transgenerational transmission of non-genetic information (parental effect) about environmental conditions is more beneficial when environmental conditions are similar between parents and offspring. The mechanism underlying these observations is currently unknown, however, and identifying it will be an interesting next step.

sRNAs are known to play an important role in mediating phenotypic plasticity across different taxonomic groups and are believed to be part of an adaptive response to fluctuating environmental conditions. In *C*. *elegans*, for example, developmental arrest caused by starvation leads to an up-regulation of endogenous siRNA and down-regulation of their mRNA targets primarily associated with nutrient storage [50]. In the thale cress, *Arabidopsis thaliana*, siRNA production is induced by salt stress and reduces the expression of a proline catabolic gene, leading to proline accumulation, which is important for salt tolerance [83,84]. Further evidence comes mostly from studies of responses to biotic stress, namely pathogenic infections in both plants and animals [85]. In plants, the role of sRNAs in silencing pathogen gene expression has been established and seems to be widespread [86,87]. In *A*. *thaliana*, cells infected with the fungal pathogen *Botrytis cinerea* secrete vesicles that deliver sRNAs into the fungus, inducing silencing of the fungal genes associated with pathogenicity [88]. In some cases, pathogen counter-defense can evolve leading to a molecular arms race between the hosts’ silencing mechanisms and the pathogens’ evasion strategies [89]. Similarly, *C*. *elegans* uses sRNA interference as an immune response to artificial viral infections, with RNAi-defective worms displaying aggravated infections while RNAi-enhanced worms inhibit the production of infectious progeny virus [90]. This reduction of pathogenic infections caused by the production of sRNAs supports the idea that sRNAs play a role in adaptation to novel environments. Furthermore, pathogenic infections are typically localized in time and space, providing the type of autocorrelated environments that we find favor transgenerational inheritance of sRNA amplification rates.

sRNAs are also known to have a number of functions in the germline and can be transmitted between generations. In *Drosophila*, piRNAs have been shown to protect the germline against the genomic replication of transposable elements by targeting transposon transcripts [91,92]. In addition, inherited germline-derived piRNAs affect the deposition of histone marks and initiation of primary piRNA biogenesis in the offspring and thereby act as an epigenetic memory across individual generations [93,94]. Similarly, in *C*. *elegans*, worms infected with different viruses (e.g., flock house virus, Orsay virus) not only develop an siRNA-based antiviral response to silence viral RNAs and stop the infection, but they also pass this response on to their offspring [49,95]. This transgenerational immunogenic memory works as a form of inherited vaccination against future infections [96]. Additionally, the inheritance of sRNAs (piRNAs and siRNAs in particular) allows the transmission of information about previous exogenous viral RNA infections in *C*. *elegans* and information about endogenous stress-related processes, like starvation. Worms derived from starved great-grandparents and kept fed for three individual generations showed a siRNA response to their previous ancestors’ starvation experience, with differentially expressed siRNAs targeting genes involved in nutrition. The transmission of sRNA levels was shown to persist for up to four generations without further stimulation [50].

While the role of sRNAs in protecting the soma and germline from environmental stressors and the transmission of sRNAs across generations is undeniable, the adaptive value of the transmission of sRNAs across generations is debated. However, recent findings in *C*. *remanei* suggest that non-genetic inheritance may be an adaptive response to unstable environments [81]. The evolution of plastic amplification rates for sRNAs might provide hosts with an edge in the race against everchanging viruses and other disease agents. Indeed, disease agents may sometimes supply the sRNA whose amplification has been co-opted for host defense [97]. Similarly, abiotic environmental factors might drive the evolution of plastic amplification processes to help organisms maintain their physiological state (homeostasis) when exposed to adverse environmental conditions. Here, we provide a proof-of-concept model for the role of environmental fluctuations in the adaptive evolution of sRNAs, considering both plastic amplification in response to current conditions and the potential for inheritance of sRNA transcripts or amplification rates. Future theoretical work would benefit from exploring stochastic dynamics and other features, such as plasticity in transcription rates as well as amplification rates. Our theoretical understanding of the roles that sRNA amplification and inheritance play will also benefit from empirical studies exploring and quantifying the exact processes that alter transcription and amplification rates within and across generations. Together, theoretical and empirical work promise to shed more light on the fitness advantages of transgenerational transmission of sRNAs as well as the mechanisms by which plastic sRNA amplification is transmitted from parents to offspring through epigenetic marks or cytoplasmic amplification agents.

## Supporting information

S1 Fig Examples of environmental scenarios with different levels of similarity between the environment of parents and offspring (*p*_*ε*_). Note that we held the number of relaxed (*ε* = 0.1) and stressful (*ε* = 0.9) environments constant (50% each) to allow us to optimize the system in the absence of epigenetic inheritance.
(TIF)

S2 Fig Fitness landscape at time t within a generation, *W*(*n_t_, ε*_*g*_,*P_b_*), as a function of the quantity of sRNA at that time (nt) and the current environmental conditions (*ε*_*g*_) with no plasticity in amplification rate (*P_b_* = 0). The following values in the fitness function were used across the study: *α* = 15.0, *β* = 0.1, *h* = 5.0, *C*_*n*_ = 10^−5^ and *C*_*b*_ = 50*C*_*n*_.
(TIF)

S3 Fig Dynamics of individual sRNA production across cell divisions under different biological conditions (parameter values). Amounts of sRNAs (*n*) reach their steady state values over time, measured in cell divisions. Solid lines show dynamics in a wild type individual (with *b* = 0; default, slow and fast dynamics), and dashed lines show dynamics in an amplifying mutant (*b*>0; default, slow and fast dynamics).
(TIF)

S4 FigProduction of sRNAs (*n*) given different transcription and degradation rates, under wildtype conditions (*b*^*-*^ = 0, *r^-^_germ_* = 0 and *P_b_^-^* = 0; left), and its geometric mean fitness (*W*_*GEO*_, right) under fluctuating environmental conditions ($\bar{\varepsilon }$ = 0.5).The black circle indicates the intrinsic degradation rate (*d* = 0.1) and optimal transcription rate (*μ*^−^ = 6.798) with $\bar{\varepsilon }$ = 0.5, as was used across the study.(TIF)

S5 Fig Invasiveness of a coupled mechanism of sRNA amplification (*b*^*+*^>0, strategy B) and transmission (*r*^*+*^_*germ*_>0, strategy C; left panel). The black point indicates the highest geometric mean fitness relative to strategy A (selection coefficient; optimal *r*^*+*^_*germ*_ = 0.1 and *b*^*+*^ = 0.013). Note that transmission makes some amplification beneficial even though amplification alone is detrimental. Solid line (left panel) indicates *s*^+^ = 0, separating selectively beneficial parameter combinations from detrimental combinations. Dashed lines indicate |*s*^*+*^|≤0.001. The red curve (right panel) shows the selection coefficient for the optimal *r*^*+*^_*germ*_ = 0.1, with a maximum at *b*^*+*^ = 0.013.
(TIF)

S6 Fig Dynamics of sRNA production across generations in strategy D when *P*_*b*_ = 1.0 with *p*_*ε*_ = 0.11 (blue curve) and *p*_*ε*_ = 0.89 (red curve). Note that production increases under conditions of high environmental stress.
(TIF)

S7 Fig Invasiveness of strategies D, E and F when the cost of plasticity (*C*_*b*_) is 500 times higher than the cost of sRNA production (*C*_*n*_), rather than 50 times as shown in Fig 4. See **Fig 4** for additional details.
(TIF)

S8 Fig Selection on strategies that amplify sRNA in response to environmental conditions (strategies D, E and F), when the dynamics of sRNA within an individual are slowed to 0.75 times the original speed, preventing sRNA levels from reaching optimal levels within one generation. See **Fig 4** for additional details.
(TIF)

S9 FigInvasiveness of modified strategies D, E and F ($P_{b}^{+}$>0) that also enable sRNA transcripts to be transmitted to offspring ($r_{germ}^{+}$>0).Black points indicate the highest geometric mean fitness relative to strategy A; in all cases, the optimum resides at $r_{germ}^{+}$ = 0.11 and $P_{b}^{+}$ = 0. Solid lines indicate *s*^+^ = 0, separating selectively beneficial parameter combinations from detrimental combinations. Dashed lines indicate |*s*^*+*^|≤0.001.(TIF)

S1 Appendix Mathematica code used to analyse the model and run the simulations. (ZIP)
