# Supplementary figures and images for: Evolution of plasticity in production and transgenerational inheritance of small RNAs under dynamic environmental conditions
Source: PLoS Genet. 2021 May 26;17(5):e1009581. doi: 10.1371/journal.pgen.1009581 (PMC8186813; doi:10.1371/journal.pgen.1009581)

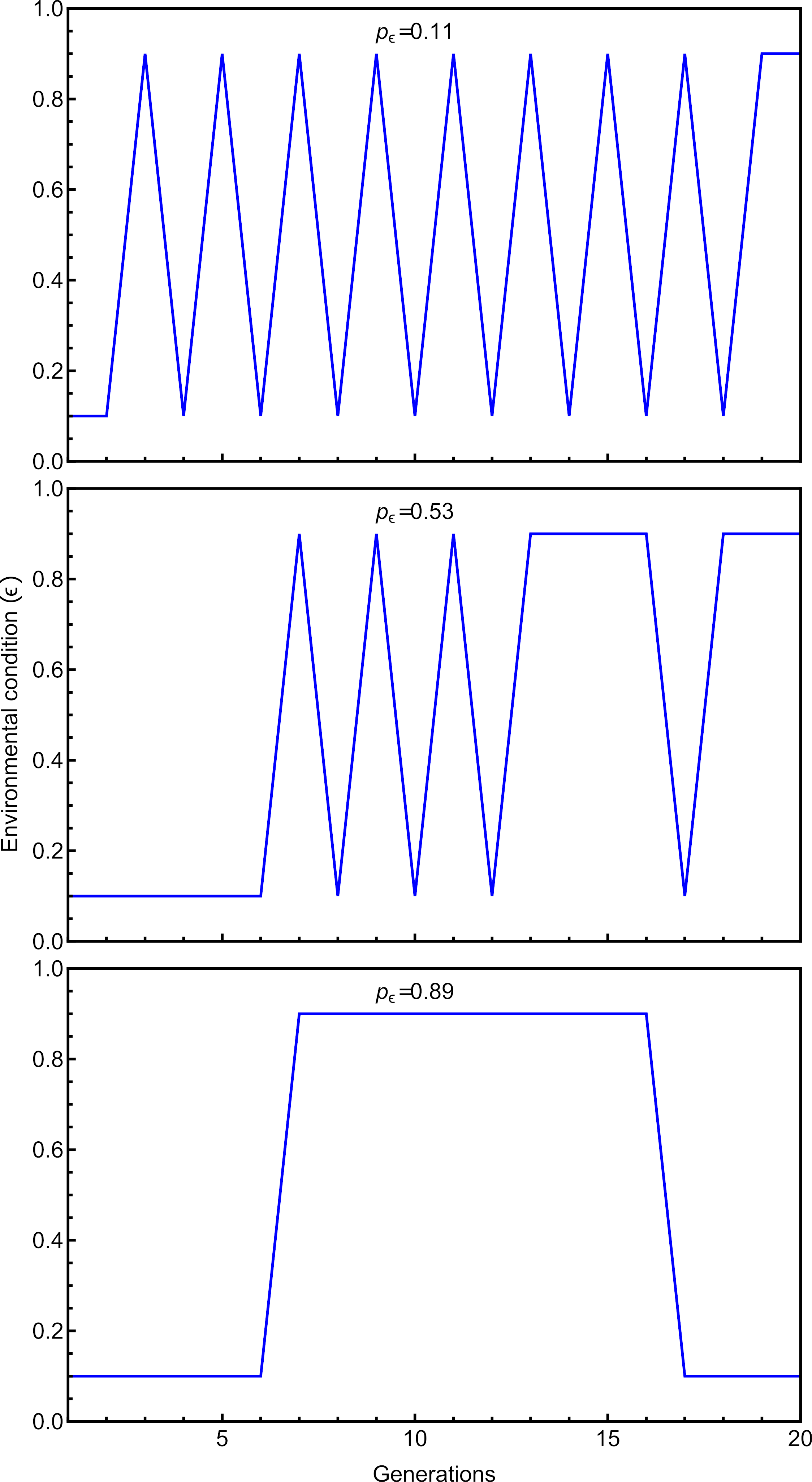

Supplement: S1 Fig — Note that we held the number of relaxed (ε = 0.1) and stressful (ε = 0.9) environments constant (50% each) to allow us to optimize the system in the absence of epigenetic inheritance. (TIF) [file pgen.1009581.s001.tif]

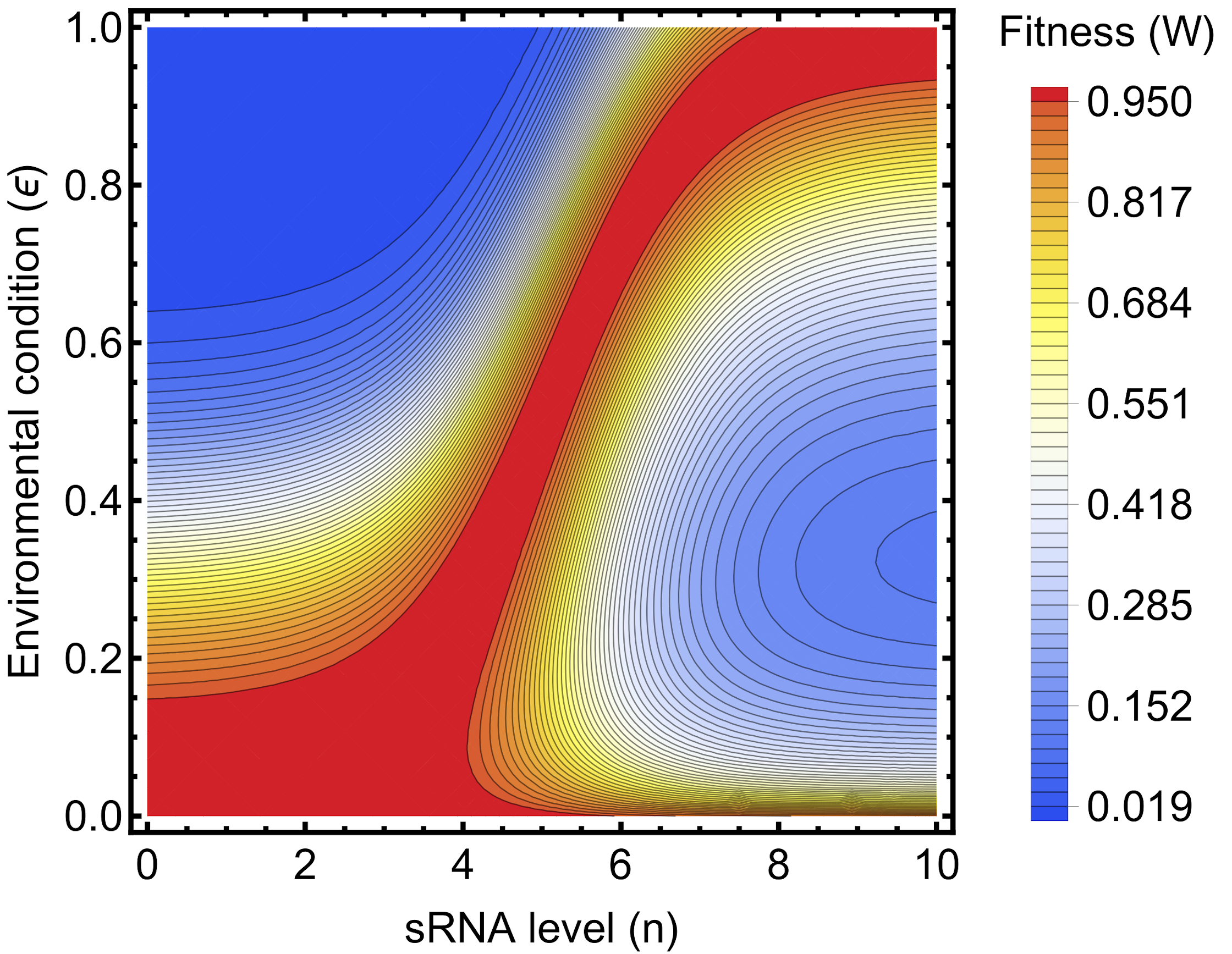

Supplement: S2 Fig — The following values in the fitness function were used across the study: α = 15.0, β = 0.1, h = 5.0, Cn = 10−5 and Cb = 50Cn. (TIF) [file pgen.1009581.s002.tif]

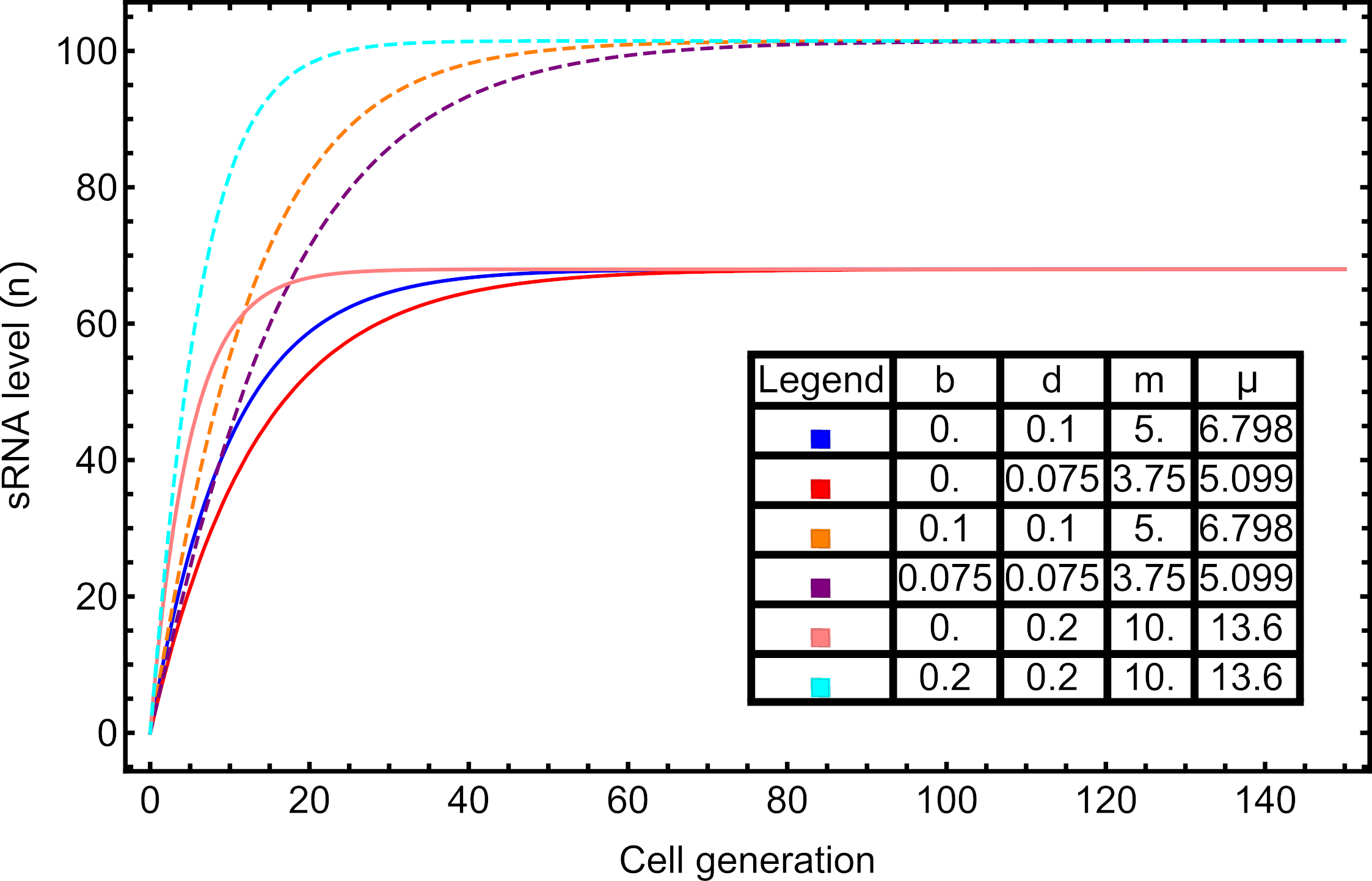

Supplement: S3 Fig — Amounts of sRNAs (n) reach their steady state values over time, measured in cell divisions. Solid lines show dynamics in a wild type individual (with b = 0; default, slow and fast dynamics), and dashed lines show dynamics in an amplifying mutant (b>0; default, slow and fast dynamics). (TIF) [file pgen.1009581.s003.tif]

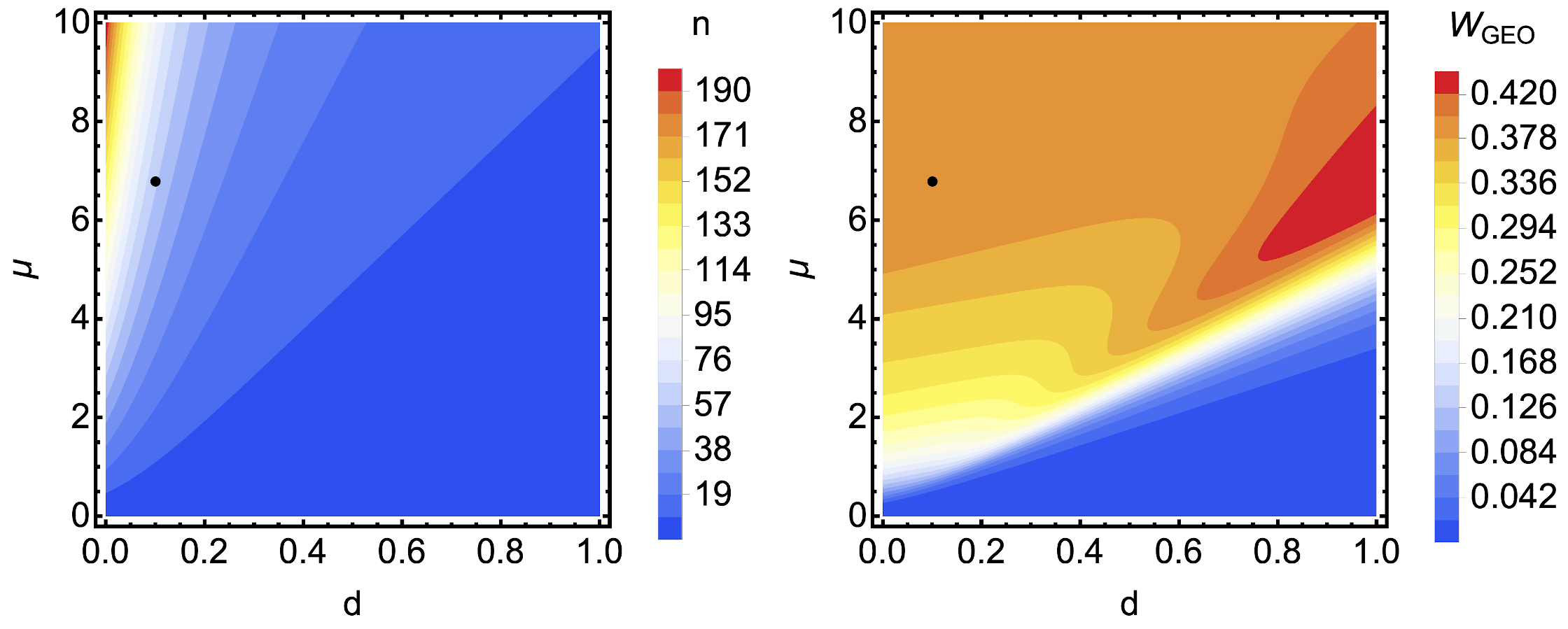

Supplement: S4 Fig — The black circle indicates the intrinsic degradation rate (d = 0.1) and optimal transcription rate (μ− = 6.798) with ε¯ = 0.5, as was used across the study. (TIF) [file pgen.1009581.s004.tif]

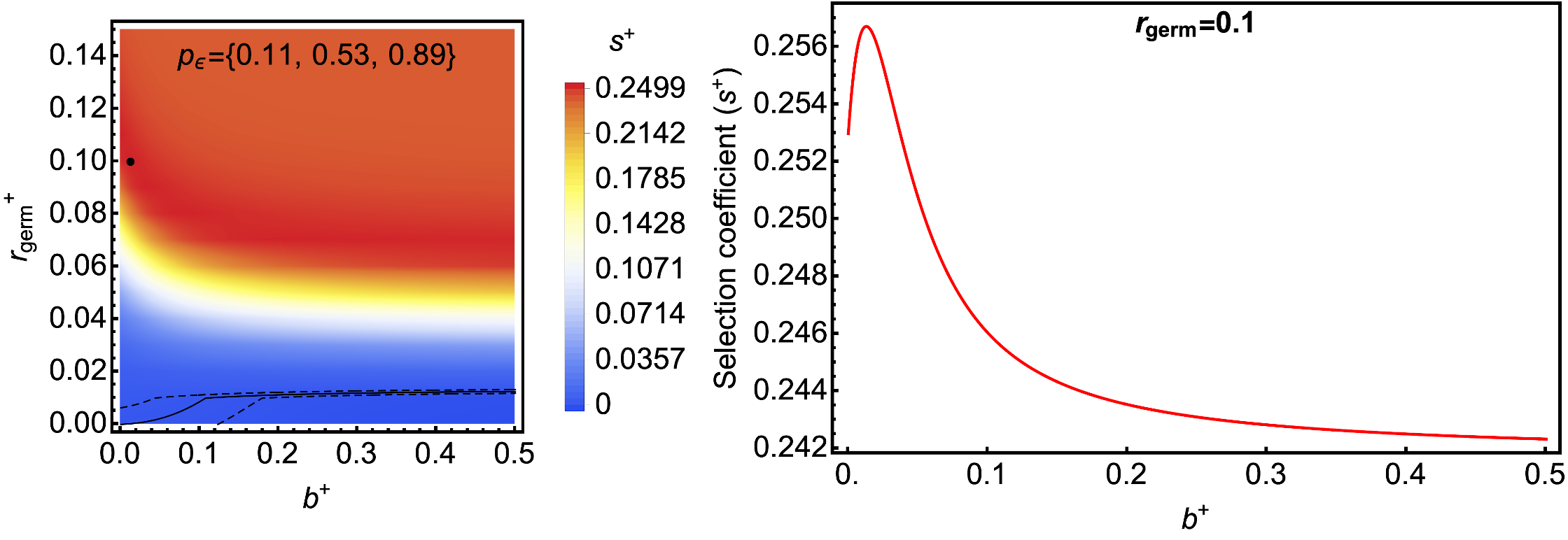

Supplement: S5 Fig — Invasiveness of a coupled mechanism of sRNA amplification (b+>0, strategy B) and transmission (r+germ>0, strategy C; left panel). The black point indicates the highest geometric mean fitness relative to strategy A (selection coefficient; optimal r+germ = 0.1 and b+ = 0.013). Note that transmission makes some amplification beneficial even though amplification alone is detrimental. Solid line (left panel) indicates s+ = 0, separating selectively beneficial parameter combinations from detrimental combinations. Dashed lines indicate |s+|≤0.001. The red curve (right panel) shows the selection coefficient for the optimal r+germ = 0.1, with a maximum at b+ = 0.013. (TIF) [file pgen.1009581.s005.tif]

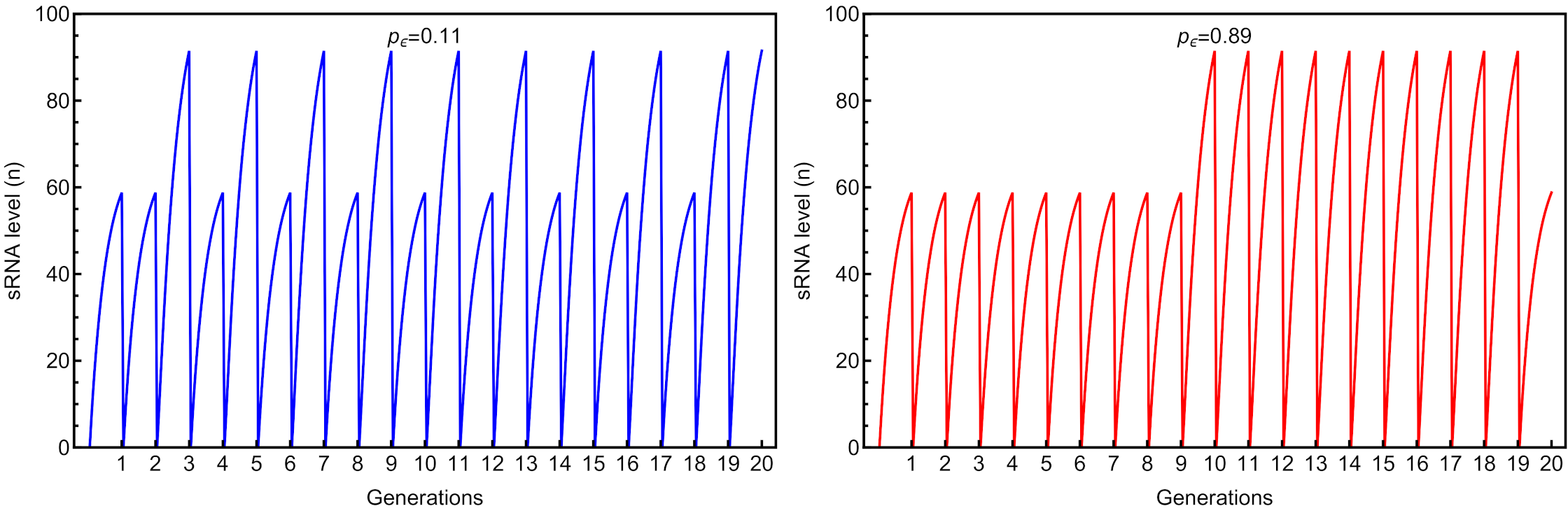

Supplement: S6 Fig — Note that production increases under conditions of high environmental stress. (TIF) [file pgen.1009581.s006.tif]

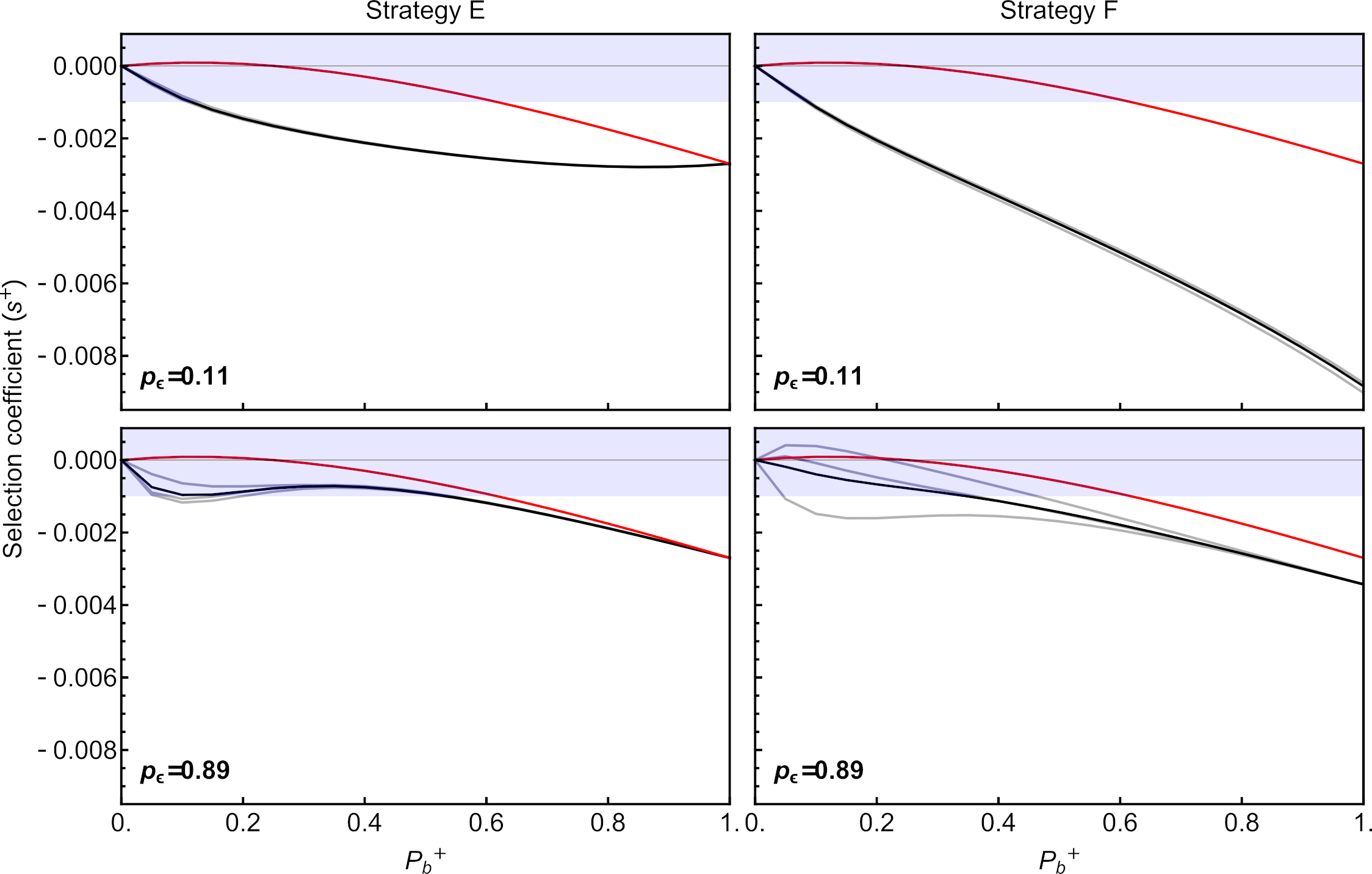

Supplement: S7 Fig — See Fig 4 for additional details. (TIF) [file pgen.1009581.s007.tif]

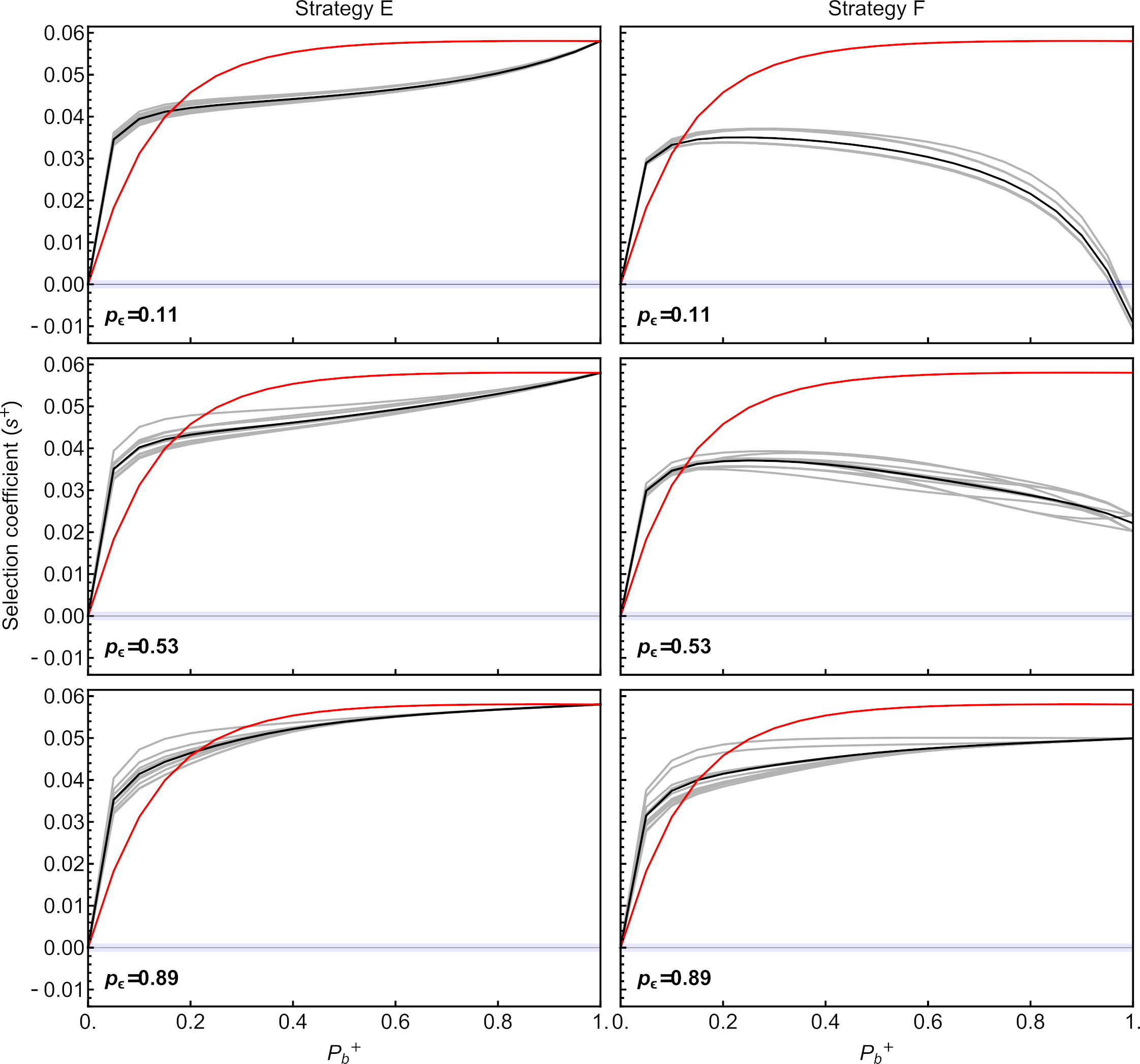

Supplement: S8 Fig — See Fig 4 for additional details. (TIF) [file pgen.1009581.s008.tif]

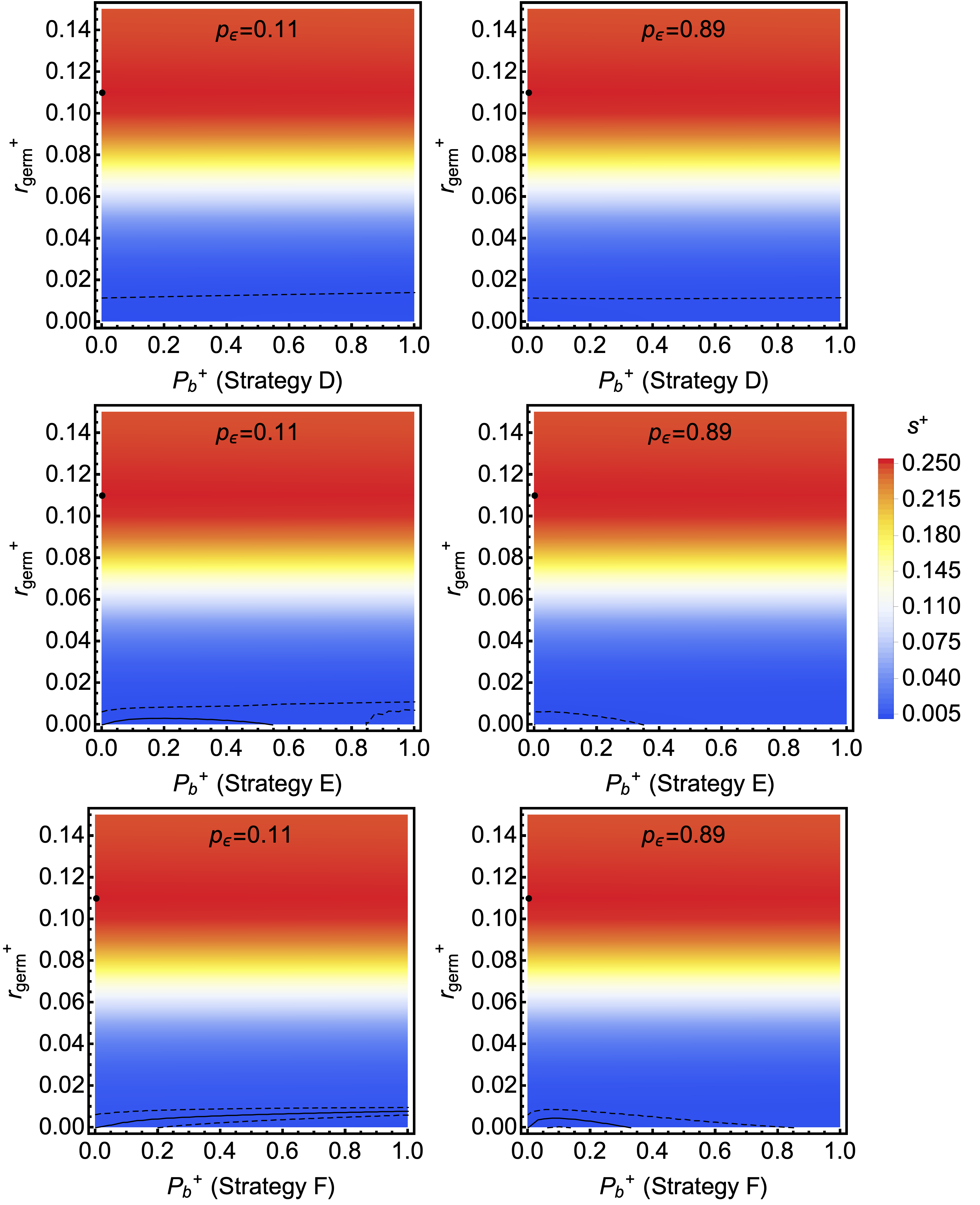

Supplement: S9 Fig — Black points indicate the highest geometric mean fitness relative to strategy A; in all cases, the optimum resides at rgerm+ = 0.11 and Pb+ = 0. Solid lines indicate s+ = 0, separating selectively beneficial parameter combinations from detrimental combinations. Dashed lines indicate |s+|≤0.001. (TIF) [file pgen.1009581.s009.tif]
